# How bodily expressions of emotion after norm violation influence perceivers’ moral judgments and prevent social exclusion: A socio-functional approach to nonverbal shame display

**DOI:** 10.1371/journal.pone.0232298

**Published:** 2020-04-30

**Authors:** Otto Halmesvaara, Ville J. Harjunen, Matthias B. Aulbach, Niklas Ravaja

**Affiliations:** 1 Department of Social Research, Faculty of Social Sciences, University of Helsinki, Helsinki, Finland; 2 Department of Psychology and Logopedics, Faculty of Medicine, University of Helsinki, Helsinki, Finland; Middlesex University, UNITED KINGDOM

## Abstract

According to a socio-functional perspective on emotions, displaying shame with averted gaze and a slumped posture following a norm violation signals that the person is ready to conform to the group’s moral standards, which in turn protects the person from social isolation and punishment. Although the assumption is intuitive, direct empirical evidence for it remains surprisingly limited and the mediating social-psychological mechanisms are poorly understood. Therefore, three experimental studies were conducted to investigate the social function of nonverbal displays of shame in the context of everyday norm violations. In Study 1, participants evaluated ten different expressions of emotion in regard to their affective valence, arousal, dominance, as well as social meaning in the context of norm violations. Displays of shame and sadness were seen as the most similar expressions with respect to the three affective dimensions and were perceived to communicate the perpetrator’s understanding of the group’s moral standards most effectively. In Study 2, participants read vignettes concerning norm violations and afterward saw a photograph of the perpetrator displaying nonverbal shame, sadness or a neutral expression. Perpetrators’ displays of shame and sadness increased perceived moral sense and amplified the observers’ willingness to cooperate with the perpetrators. However, neither display weakened the observer’s willingness to punish the perpetrator. In Study 3, the perpetrator was shown to display shame, sadness, anger or a neutral expression after getting caught at mild or severe norm violation. The results replicated previous findings but revealed also that the social effects of shame and sadness displays on punitive and cooperative intentions were mediated by different social appraisals. For example, display of shame uniquely reduced punitive intentions by increasing the perpetrator’s perceived moral sense, whereas expressions of both shame and sadness evoked empathy in the observers, which in turn reduced the punitive intentions. These results give support to the assumption that nonverbal shame displays serve a unique social function in preventing moral punishment and social exclusion. However, this support is only partial as the social functions of displaying shame are largely parallel to those of expressing sadness in the situation.

## Introduction

For many decades, the feeling of shame has intrigued clinical psychologists and researchers alike. Much ink has been spilled over questions such as, what are the clinical and mental health implications for experiencing shame, how this experience differs from other closely related emotions such as guilt and embarrassment, and whether the feeling of shame motivates adaptive or maladaptive social behaviors [1–3]. The focus of these reflections has largely been on the individual who experiences shame [4]. Much less attention has been paid to the possibility that shame has an important communicative role in regulating the social behavior of dyads and of larger groups (e.g., [5]). As with many other emotions, the social functions of shame are argued to operate through nonverbal displays manifested in facial cues, gestures, and postures [6, 7]. In the current research, we examined the social functions of displaying shame after norm violations by investigating the social appraisals and behavioral intentions of persons witnessing norm violations and the perpetrator’s bodily expressions of emotion.

So far, the few studies that have empirically scrutinized the socio-functional role of shame demonstrated that expressing shame verbally after a norm violation elicits positive evaluations of the perpetrator’s moral sense [8, 9] and increases readiness to forgive as well as decreases willingness to punish the wrongdoer [10, 11]. However, less is known about the implications of nonverbal displays of shame. The lack of research in this area is a serious deficiency, especially from a socio-functional perspective, since nonverbal expressions of emotion are assumed to play a crucial role in regulating the social behavior of individuals and groups [6]. The scarcity of research is also surprising because a specific set of nonverbal bodily cues, including avoidance of eye contact and a slumped posture, has been consistently associated with the feeling of shame in divergent cultural settings [4, 12–14]. In fact, even congenitally blind athletes from different cultural backgrounds have been shown to avoid eye contact and slump in response to failure or defeat [15]. Moreover, comparative research has revealed noticeable similarities between human nonverbal shame displays and other social mammals’ way of appeasing dominant individuals [16].

The cross-cultural uniformity of bodily manifestations of shame among humans and the similarities between species hint that displaying nonverbal shame has an adaptive role in interpersonal relations. Accordingly, Fessler [17] suggested that there are two major social functions of shame expression. The first of these, which is shared with many other primate species, is to express submissiveness to individuals higher in the dominance hierarchy. The main purpose here is to prevent unnecessary and costly social and physical conflicts with those higher in social rank. The other, socially more sophisticated and phylogenetically more recent, function is to seek approval from others and to prevent social exclusion. Expressing shame after norm violations signals that the perpetrator understands that s/he has violated the rules of the community and is ready to conform to the group’s standards, and this, in turn, should indicate to others that s/he is still a potentially valuable cooperation partner and a fully intergraded member of the group.

These two suggested functions are intuitively compelling but direct empirical support for them has remained surprisingly scarce. In fact, in a review by Martens et al. [14], only one published study was identified in which the functional effects of nonverbal shame and other emotional displays were experimentally tested. The participants in the study [18] evaluated a hypothetical situation in which a student had made a mistake during their presentation and afterward displayed either nonverbal shame, embarrassment, anger or amusement, or took a neutral stance. A picture of nonverbal shame (averted gaze and head tilted downwards) elicited more empathy towards the student than any other expressed emotion, which was interpreted as giving support to the unique appeasement effect of shame display. More recently, Hornsey et al. [19] examined the effects of “embodied remorse” (kneeling and crying) in the context of public apology. The authors found that the apologizer’s nonverbal signs of remorse created a more favorable impression of them when compared to a situation in which no remorse was shown. Although embodied remorse does not directly correspond to shame displays, there are arguably strong similarities in the submissive body language between the two displays, for which reason the results may be informative about the social functions of nonverbal shame that are the topic here.

Existing studies on nonverbal shame are nevertheless limited. The two studies conducted so far rely on diverse theoretical frameworks, compare different bodily displays, and do not sufficiently consider how the functions of shame display differ from those of other closely related emotional expressions. Moreover, even though nonverbal shame would have a distinct social function, it remains unanswered what kind of socio-cognitive mechanisms are driving it and whether the functions appear only in specific contexts, such as after moral standards have been violated. Therefore, to bolster the evidence for the adaptive functions of shame display, further empirical studies are called for. In their review, Martens et al. [14] suggested the social functionality of nonverbal signs of shame should be examined by comparing it to the social consequences of other closely related expressions, such as the expression of sadness. Indeed, just like shame, sadness is also an inherently negative emotion with a submissive social orientation but is related to distinct and easily recognized body language. Therefore, it would be necessary to experimentally compare influences of the two expressions on observers’ social appraisals and behavioral intentions, specifically in the context of norm violation, to find out whether appeasing social influence is uniquely evoked by nonverbal displays of shame. Alternatively, it is possible that multiple expressive configurations evoke similar social behaviors in the observers. In this case, it would be important to examine whether there are shared elements of nonverbal manifestation resulting in similar social appraisals.

What comes to socio-cognitive mechanisms, it seems that there are at least three potential driving forces that could mediate the appeasing effect of shame displays. The first of these can be derived directly from Fessler’s [17] model mentioned above. Fessler argued that shame displayed after a norm violation signals that the perpetrator understands what s/he did was wrong, which implies to others that s/he is still a valuable group member. Thus, it can be hypothesized that the effects of nonverbal shame on observers’ willingness to cooperate and/or punish the perpetrator depend on the observers’ evaluations of perpetrators’ moral sense.

Another potential mediator is empathy. Empathy belongs to the family of compassion-related emotional states that arise as a reaction to the suffering of others [20]. Given that shame is a highly unpleasant and distressing emotion, seeing somebody ashamed is likely to elicit feelings of empathy and thus to weaken punitive intentions. Moreover, the relationship between empathy and appeasement could also be extended to cooperation, since it has been suggested that feelings of empathy initiate and maintain cooperative relationships [20, 21]. In the context of nonverbal displays of shame, this would mean that empathy mediates the effects of nonverbal shame on subsequent cooperative as well as punitive behaviors toward the perpetrator.

The third potential mediator that should be considered is perpetrators’ perceived social anxiety. In a study by Stearns and Parrott [8] verbal expression of shame in the context of norm violation was linked to heightened evaluations of transgressors’ social anxiety. Moreover, social anxiety has been described as an important aspect of subjective experiences of shame [1]. Believing that the perpetrator feels social anxiety after violating a norm indicates to the observer that the perpetrator is worried about how others might see him/her, which, in turn, could implicate the perpetrator’s potential as a reliable group member. Therefore, we believe that perceived social anxiety could also work as a proximate mechanism to convey the functional effects of nonverbal shame on cooperation and punishment.

As described in the present article, a series of three studies were conducted to investigate the behavioral intentions and moral judgments evoked by nonverbal shame displays (operationalized as slumped posture with downward head tilt and downward eye gaze); how the effects of shame displays differ from those of other closely related emotional expressions; and finally, what kind of mediating mechanisms are involved in these effects. In Study 1, participants were asked to rate a large number of nonverbal expressions and indicate to what extent each expression signaled that the person had understood their behavior to be wrongdoing and was ready to conform to the group’s standards. Then, in Study 2, the effects of nonverbal shame displays and the other closely related emotional expressions were experimentally compared to see if the display of shame uniquely decreased observers’ intentions to socially exclude and punish the perpetrator as suggested by Fessler [17]. In Study 3, the effect of nonverbal shame on observers’ social exclusion and intentions to punish a perpetrator was compared to one phenomenologically similar and to one opposite nonverbal expression in the context of both more and less severe moral transgressions. Here, we also investigated whether and how the perpetrator’s perceived social anxiety and moral sense, as well as empathy felt towards him/her, mediated the link between shame display and observers’ cooperative and punitive intentions.

## Study 1

### Aims

The socio-functional account assumes that nonverbal displays of shame should have unique qualities not shared by other nonverbal expressions. Namely, bodily shame displays are assumed to be the most effective nonverbal expression to convey an understanding of one’s wrongdoing and readiness to grow humble in the situation [17, 18]. To empirically test this uniqueness assumption, we first identified emotional expressions that have similar affective features as shame displays by asking the participants to rate nine expressions of basic and self-conscious emotions, and a neutral expression, on three affective dimensions: emotional valence, arousal, and dominance. Six most commonly used basic emotions (sadness, anger, fear, surprise, happiness, disgust [22]) and all of the self-conscious emotions with a validated nonverbal expression (shame, embarrassment, pride [23]) were used, along with a neutral expression, to probe similarities and differences between separate emotion categories. In their review, Martens et al. [14] suggested that comparing displays of shame to that of sadness could be particularly informative since both are intense emotions with negative valence and a submissive social orientation. Therefore, we hypothesized (H1) that expression of sadness would be seen as more similar to shame displays in terms of their affective dimensions (i.e., valence, arousal, and dominance) than other emotional expressions. However, based on the socio-functional account [17, 18] and earlier empirical evidence on verbal expressions of shame [8, 9], displaying shame should be uniquely effective in restoring observers’ view of the perpetrator's moral sense. Therefore, we hypothesized (H2) that the display of shame would be seen as the most effective means to signal understanding of one’s wrongdoing (i.e., moral sense) and readiness to conform to the group’s standards after a norm violation (i.e., conformity).

## Method

### Participants

The participants were recruited through student organization mailing lists at the University of Helsinki. One hundred and thirty participants (M_age_  = 26.48; SD = 6.11) decided to take part in the experiment. There were no restrictions for participation and all willing participants were included in the sample. The sample consisted of 102 women, 19 men and 9 persons who selected “other or preferred not to say.” Before giving informed consent, participants were informed of the anonymized nature of their responses and their right to withdraw from the study at any time. The PANGEA program [24] was used to determine the sample size required to detect a medium-sized effect (Cohen’s d = 0.40) of emotional expression with 80% statistical power and .05 alpha level. PANGEA has been developed for power analysis of general ANOVA designs. It can handle several factors, each with any number of levels and is thus routinely used in experimental psychology (e.g., [25–27]). When a 1 x 10 (10 emotional expressions) within-subject design was applied, 130 participants granted 80% power to detect the expected effect. Therefore, the decision to proceed was made.

### Procedure

Participants were recruited to take part in an online experiment in which they were asked to evaluate a series of photographs of emotional expressions based on three affective dimensions and social appraisals. After reading the instructions and entering information about their gender and age (step 1), participants were shown an emotional expression image (330 x 380 px, self-timed) of a white man or woman (step 2) and asked (step 3) to evaluate the expression on dimensions of emotional valence (e.g., pleased vs. unpleased), arousal (e.g., relaxed vs. tense), and dominance (submissive vs. dominant), respectively. The scales are described in detail in section 2.2.4. After giving their responses, participants were asked to move forward to the next phase (step 4) using a button press. Upon this response, participants saw an image of the same model with same expression again and were instructed to imagine that the person in the image was disapproved of by others after getting caught violating society’s moral standards (i.e., the participants were asked to imagine a general situation of norm violation but no specific scenario was presented). Participants were asked (step 5) to evaluate the perpetrator based on three social appraisals: their moral sense, their readiness to conform to moral standards after the violation, and appropriateness of their emotional expression, respectively. The trial procedure was repeated ten times, once for each expression category (shame, sadness, fear, disgust, anger, embarrassment, neutral, surprise, happiness, and pride), the order of expressions being randomized between participants. Participants saw the same model throughout these repetitions; the choice of model (photographs of one woman and man model were used in the study) was randomized between participants. Finally (step 6), participants were again shown each expression, one at a time (in a random order) and asked to select the emotion category (from a list of ten categories) that best depicted each of the model’s expressions. (See Measures section for further details of the emotion recognition measure.) The entire procedure was presented online using the Psytoolkit program (www.psytoolkit.org).

### Stimulus material

**Fig 1:**
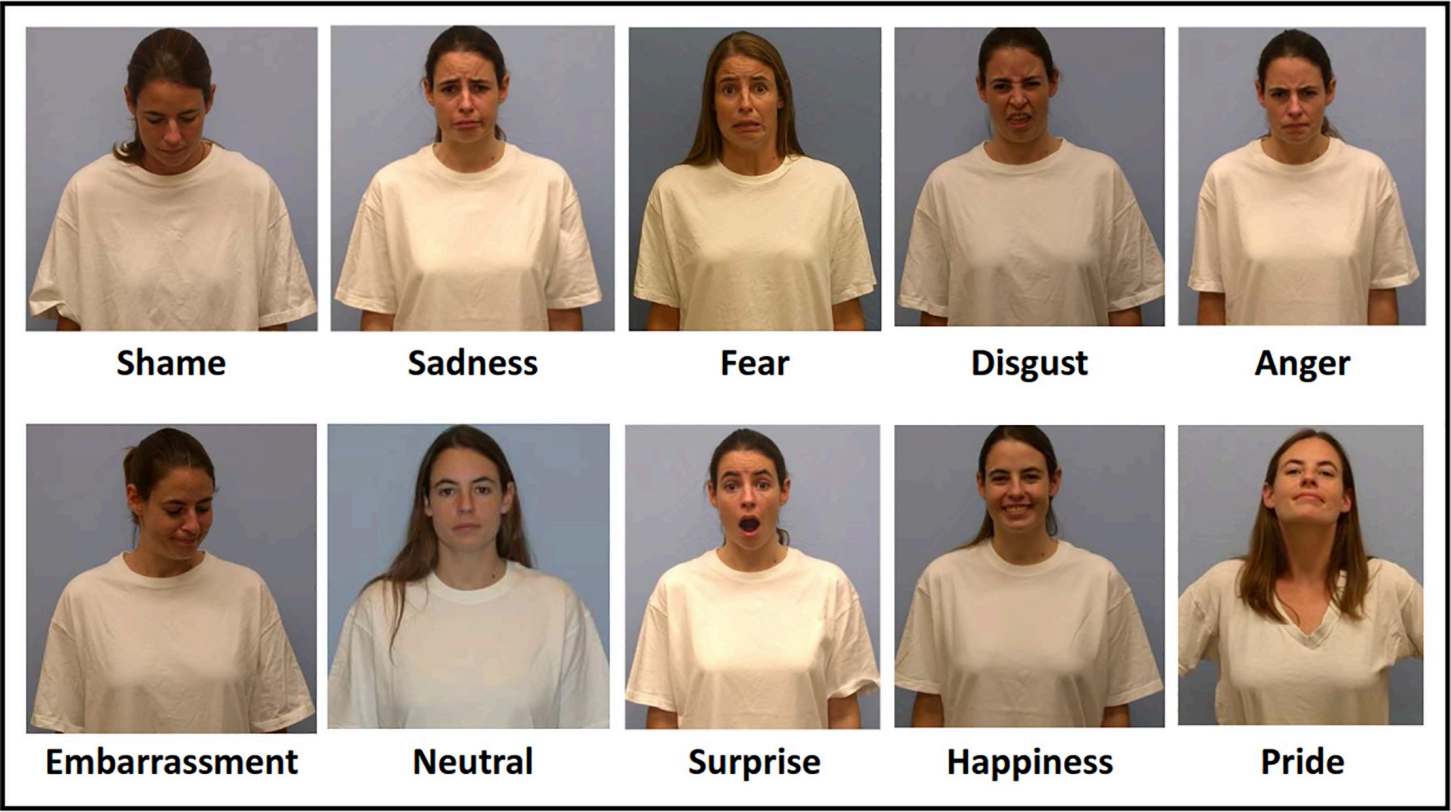
Expressions of basic and self-conscious emotions used in Study 1. The expression pictures were adopted from the UC Davis set of emotional expressions (UCDSEE; [23]) with the permission of the copyright holder. Dimensions of the pictures have been adjusted for the current presentation.

The pictures of a white man and woman were taken from the UC Davis set of emotional expressions (UCDSEE; [23]). UCDSEE is a FACS-verified and empirically validated set of 9 emotional expressions (shame, sadness, fear, disgust, anger, embarrassment, surprise, happiness, and pride) and a neutral expression displayed by female and male, white and black models [23]. The set has previously been successfully used in numerous studies (e.g., [14, 28]. Contrary to other commonly used sets of expression pictures, the UCDSEE includes a selection of emotions that critically involve the person’s self-image, also called “self-conscious emotions” [29]. Moreover, the expressions include not only facial action units but also head orientation and the posture of the model’s torso. The UCDSEE images are available in the following open repository: http://ubc-emotionlab.ca/research-tools/nucdsee/. The emotional expressions have also been presented in Fig 1. Only the expressions of white models were selected as the set of expressions was incomplete in black models.

### Measures

#### Valence, arousal, and dominance of the perpetrator’s emotional expression

The participants rated each expression picture on three affective dimensions (valence, arousal, and dominance) using the Self-Assessment Manikin (SAM) -scale, which is a pictorial assessment technique used to measure valence, arousal, and dominance [30]. The scale was modified slightly to fit the task at hand: instead of evaluating what the participant himself/herself felt, participants were asked to evaluate what the person in the emotional expression picture was feeling using the SAM -figures. In addition to the pictorial figures, the scale was anchored with text references at either end. Valence ranged from 1 (“unhappy, unsatisfied”) to 9 (“happy, satisfied”), arousal from 1 (“calm, drowsy, still”) to 9 (“energetic, agitated, alert”), and dominance from 1 (“being at the mercy of the situation, unable to control the situation”) to 9 (“being completely in control of the situation, able to control the situation”). The SAM -figures are available in the following open repository: http://irtel.uni-mannheim.de/pxlab/demos/index_SAM.html.

#### Perceived moral sense and conformity of the perpetrator

The participants were asked to evaluate the perpetrator’s moral sense (“The person understands that what s/he did was wrong”) and conformity (“The person is willing to accept the consequences of his/her actions”) on a nine-point Likert scale (1: “not at all”, 9: “very much”). Before answering the items, participants were asked to imagine that the person in the expression picture had violated society’s moral standards and was being disapproved of by others.

#### Appropriateness of the perpetrator’s expression

Finally, in the context of every emotional expression, participants were asked to rate to what extent it was socially appropriate to show a given expression after getting caught violating society’s moral standards (“The person’s reaction is appropriate in the situation”). The item was rated on a nine-point Likert scale (1: “not at all”, 9: “very much”).

#### Emotion recognition

Emotion recognition was measured in the end of the experimental session with a forced-choice item asking participants to choose out of ten categories (anger, contempt, disgust, embarrassment, fear, happiness, pride, sadness, shame, surprise, neutrality) the term that best applies to each of the model’s expressions.

### Analysis and design

The study followed a one-way repeated measures experimental design with emotional expression (shame, sadness, fear, disgust, anger, embarrassment, neutral, surprise, happiness, and pride) as a factor. A repeated measures ANOVA with expression as factor was conducted separately for each outcome measure. The outcome measures were perceived valence, arousal, and dominance of perpetrator, perceived moral sense and conformity of the perpetrator, and appropriateness of the perpetrator’s expression. Normality of residuals was visually inspected and a Greenhouse-Geisser correction was applied when the sphericity assumption of repeated measures ANOVA was violated. (Normality plots available in OSF repository: https://doi.org/10.17605/OSF.IO/B5KYM.)

## Results

### Emotion recognition

All of the emotion expressions were recognized better than chance level alone (i.e. 8.33%), with an average recognition rate of 74.85%. Shame was recognized correctly in 66.15% of cases, sadness in 79.23%, fear in 62.31%, disgust in 54.62%, anger in 92.31%, embarrassment in 36.15%, neutral in 88.46%, surprise in 94.62%, happiness in 85.38%, and pride in 89.23% of cases, respectively.

### Perceived valence, arousal, and dominance of the emotional expressions

Three separate one-way repeated measures ANOVAs were conducted setting emotional expression (shame, sadness, fear, disgust, anger, embarrassment, neutral, surprise, happiness, and pride) as a factor and perceived valence, arousal, and dominance as dependent variables. Significant differences between emotional expressions were found in the dimension of valence, *F*(6.77, 873.16) = 464.92, *p* < .001, *η*_G_^2^ = .74.

**Fig 2:**
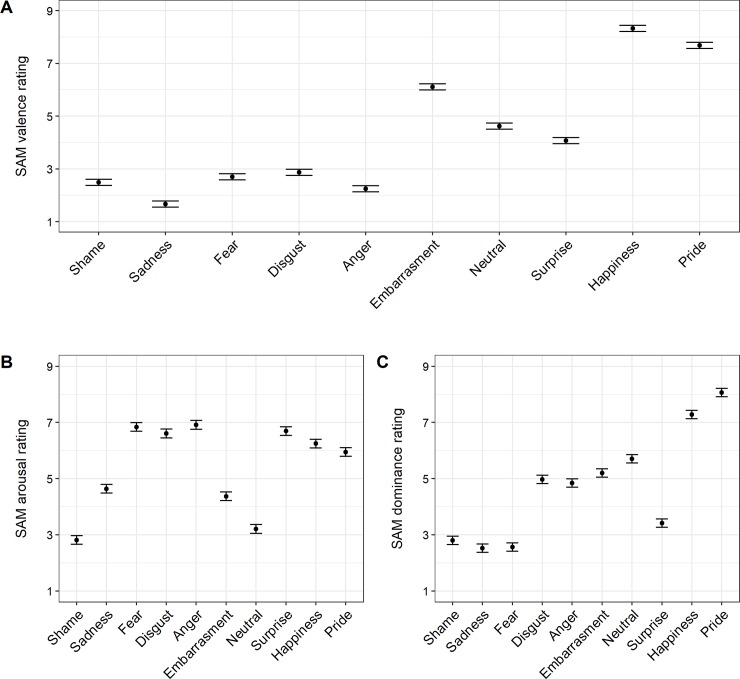
SAM Valence (A), arousal (B) and dominance (C) ratings of ten emotional expressions. Error bars reflect 95% confidence intervals of the estimated marginal means.

As can be seen from Fig 2A, shame display was perceived as more negative than expressions of disgust, embarrassment, surprise, happiness, pride, and neutral state, *t*s(1161) ≤ - 2.48, *p*s < .05). There were no significant differences in valence between shame and anger and fear, *t*s(1161) ≥ -1.39, *p*s > .12, but the expression of sadness was perceived as more negative than shame display, *t*(1161) = 5.31, *p* < .001.

Ratings of arousal likewise varied between emotional expressions, *F*(6.76, 871.61) = 122.04, *p* < .001, *η*_G_^2^ = .41. As shown in Fig 2B, shame display was perceived to depict the lowest level of arousal as compared to all other expressions, *t*s(1161) ≤ -1.98, *p*s < .05. Other low-arousal expressions were sadness, embarrassment, and neutral display. Expressions of anger, fear, and disgust had the highest arousal ratings followed by surprise, happiness, and pride. Perceived dominance likewise differed between emotional expressions, *F*(7.37, 950.66) = 192.32, *p* < .001, *η*_G_^2^ = .54. Expressions of shame, sadness and fear were perceived as the most submissive ones (see Fig 2C) and their means did not significantly differ from each other, *t*s(1161) ≥ -1.40, *p*s > .16. As compared to all other expressions, shame was perceived as most submissive, *t*s(1161) ≥ 3.12, *p*s < .01.

Taken together, ratings of submissiveness and arousal give partial support for H1, according to which the expression of sadness was seen similar to shame display in terms of affective dimensions (as compared to other expressions). However, display of sadness was perceived as more negative than display of shame, which was in disagreement with H1.

### Effects of perpetrators’ emotional expressions on perceived moral sense, conformity, and appropriateness of the reaction

To test whether perpetrators’ shame displays communicated understanding of one’s wrongdoing (i.e., perceived moral sense) and readiness to accept the consequences of their actions (i.e., perceived conformity) more effectively than other emotional expressions (H2), two one-way repeated measure ANOVAs were conducted with emotional expression as a factor and perceived moral sense and conformity as measures. Emotional expressions were found to differ in their perceived moral sense, *F*(6.87, 886.02) = 151.47, p < .001, *η*_G_^2^ = .47, as well as conformity, *F*(6.56, 845.66) = 117.65, *p* < .001, *η*_G_^2^ = .42.

**Fig 3:**
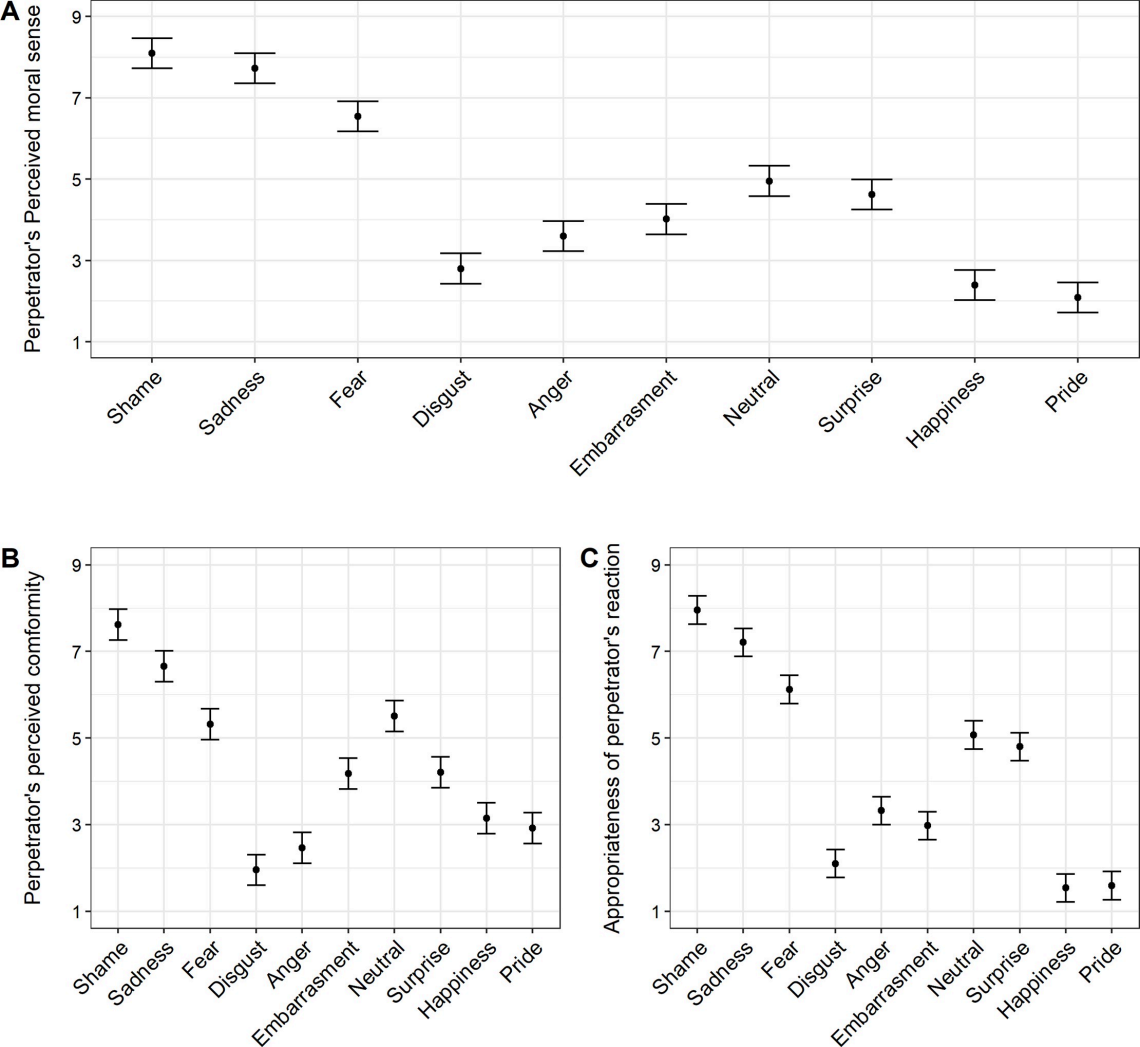
Ratings on perpetrators’ perceived moral sense, conformity, and appropriateness of their reaction. Error bars reflect 95% confidence intervals of the estimated marginal means.

As shown in Fig 3, moral sense was assessed to be higher in perpetrators displaying shame and sadness than those expressing other emotions, *t*s(1161) ≥ 6.26, *p*s < .001. However, shame and sadness did not significantly differ from each other, *t*(1161) = 1.50, *p* = .14, which was not in line with H2. However, in support of H2, perpetrators expressing shame were rated higher in their readiness to conform than perpetrators showing any other expression, *t*s(1161) ≥ 3.97, *p*s < .001.

Finally, we tested which of the expressions were considered to be the most appropriate reactions to one’s own wrongdoing. Expressions significantly differed in their perceived appropriateness, *F*(6.15, 792.91) = 228.05, *p* < .001, *η*_G_^2^ = .58. Again, display of shame was perceived as a most appropriate reaction of the perpetrator as compared to any other expression, *t*s(1161) ≥ 3.43, *p*s < .001.

## Summary study 1

All of the emotional expressions were recognized on a better-than-chance level. When different expressions were compared in regard to their ratings on three affective dimensions (valence, arousal, and dominance), expression of shame shared the most commonalities with the expression of sadness, being rated low in arousal and dominance as well as having negative valence, although sadness was perceived as more negatively valenced than shame. Display of shame was seen as the most appropriate reaction after a norm violation, and most suited to communicate that the perpetrators accepted the consequences of their actions. Shame and the expression of sadness fared equally well when perpetrators’ moral sense was evaluated. These results lend partial support for the notion that the display of shame has a unique function in communicating submissiveness and conformity following a norm violation but they also show that there are many commonalities between displays of shame and sadness in how the perpetrator is seen.

## Study 2

### Aims

After finding support for the assumption that shame displays effectively signal moral sense and conformity to the group’s standards, we examined whether the effects of displaying shame extend to observers’ moral judgments and behavioral intentions more generally. In previous studies, verbal expressions of shame have been shown to elicit more positive evaluations of perpetrators’ moral sense and indicate that a perpetrator is more socially anxious as compared to situations in which the perpetrator is not expressing any emotion [8, 9]. Nonverbal shame, in turn, has been shown to evoke empathy towards the perpetrator [18]. Based on these findings and our observations of Study 1, which illustrated the communalities between nonverbal displays of shame and sadness, we hypothesized (H3) that displaying shame after a norm violation would produce higher ratings of empathy toward the perpetrator, more favorable evaluations of perpetrators moral sense, and that the perpetrator would be seen as more socially anxious, as compared to situations in which sadness or neutrality was expressed after a norm violation. Contrasting perpetrators displaying shame to those expressing sadness was thought to inform whether and how nonverbal shame differs from expressions of other closely related emotional states [14].

In regard to behavioral intentions of observers, Hareli and Eisikovits [10] have shown that verbal expressions of shame increase readiness to forgive the wrongdoer. In addition, multiple theoretical accounts and field observations suggest nonverbal shame to appease observers by reducing their willingness to punish the perpetrator [11, 17]. Building on these findings, and following a theoretical consideration that nonverbal shame signals the cooperative potential of the transgressor [17], we hypothesized (H4) that seeing the perpetrator express shame leads to decreased willingness to punish the perpetrator and to increased willingness to cooperate with them as compared to a situation in which sadness or neutrality was expressed.

## Methods

### Participants

A total of 60 participants (*M*_age_  = 26.88, *SD* = 6.36) were recruited from the University of Helsinki central campus while ensuring equal gender distribution. There were no criteria for participation. Recruitment occurred in library lobbies and other public spheres of the campus asking passersby if they would be willing to take part in a short psychological study on emotions in exchange for one cinema ticket. The self-selected sample consisted of 29 men and 30 women and one who selected “other or preferred not to say.” Before giving informed consent, participants were told that their responses would be fully anonymized and that they would have the right to withdraw from the study at any time. *PANGEA* software [24] was used to determine the sample size required to detect a medium-sized (Cohen’s *d* = 0.40) effect of emotional expression with 80% statistical power and .05 alpha level. When 1 x 3 within-subject design with one replicate (each expression was displayed twice, once by a woman and once man actor) was applied, a sample of 58 participants granted 80% power to detect the expected medium size effect. Consequently, the target sample size was set to 60 participants.

### Procedure

**Fig 4:**
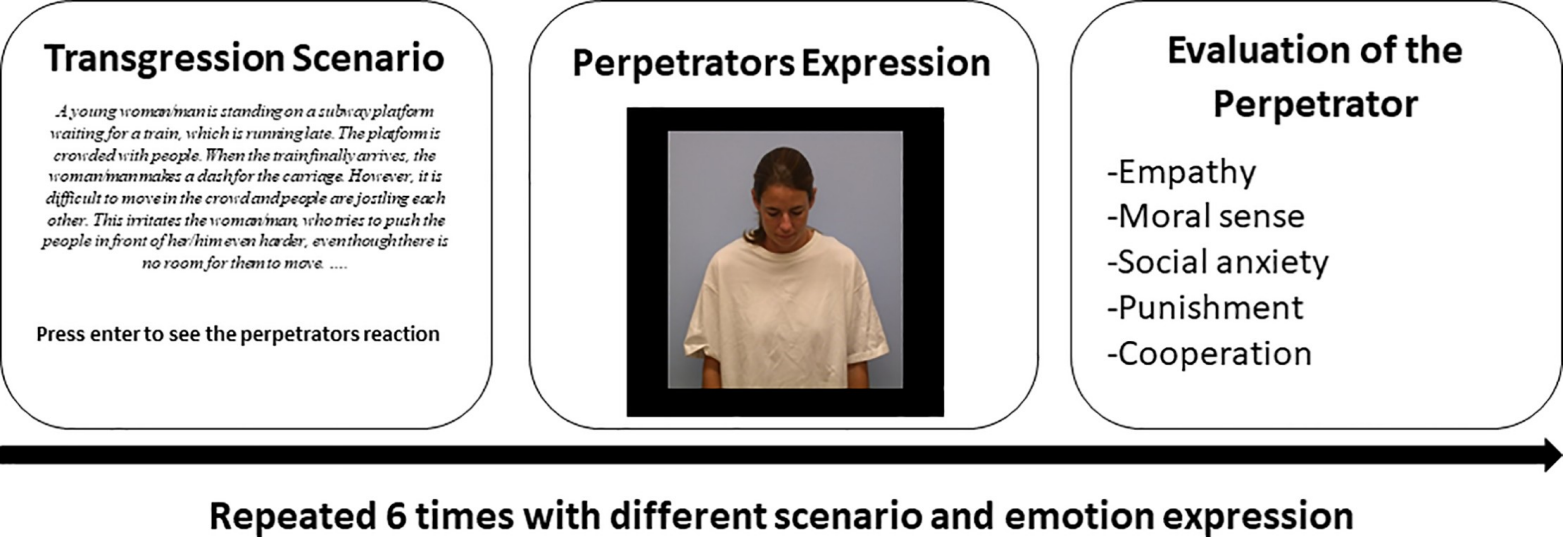
Flowchart of Study 2 trial structure.

Each participant was given a brief description of the study and was then directed to a laboratory room with a laptop computer on which to carry out the task. The task proceeded as follows. Participants read a scenario in which a young man or woman committed a mild norm violation, after which a picture of the perpetrator's emotional expression (377×457 px) on a black background was shown. The expression remained on the screen for six seconds. Next, participants were asked to rate the perpetrator according to five different measures: the empathy they felt toward the perpetrator, their inclination to punish him/her, their willingness to cooperate with the perpetrator, a scale measuring the perpetrator’s moral sense and one measuring the amount of social anxiety the perpetrator was perceived to feel in the situation, in respective order. The task was repeated 6 times with different transgression scenarios while systematically varying (according to balanced block design) the perpetrator’s gender (man vs. woman) and expression (shame, sadness, or a neutral state) across trials. Each emotional expression was displayed by both a male and a female model, the order of emotions, genders and scenarios being randomized between participants. Gender as well as the scenarios were treated as random factors to increase generalizability of the results to a wider set of contexts. However, since there were no hypotheses regarding how the perpetrator’s gender would influence or interact with the influence of emotional expression, the effect of gender was not statistically tested but was controlled through the counterbalancing procedure. See Fig 4 for the Study 2 trial structure.

### Stimulus material

#### Transgression scenarios

First, a set of 12 accounts of everyday norm violation were produced, which an independent jury of eight adults (students and staff members, randomly recruited from the university campus; *M*_*age*_*  =* 30.25 ± 2.93, 2 women) was asked to evaluate in regard to their reprehensibility (Did the person break society’s [formal or informal] rules?) and plausibility (Whether they could imagine a similar event occurring in everyday life). Based on the jury’s evaluation, a set of six scenarios (each approximately 100–150 words long) was selected for utilization in the study. One sample scenario for a male perpetrator is presented below (scenario texts and subsequent images were always matched for gender):

“A young man is standing on a subway platform waiting for a train, which is running late. The platform is crowded with people. When the train finally arrives the man makes a dash for the carriage. However, it is difficult to move in the crowd and people are jostling with each other. This irritates the man, who tries to push the people in front of him even harder, even though there is no room for them to move. The pushing makes the person in front of the man lose balance and fall over. Other passengers look disapprovingly at him.”

(See S1 Appendix for all scenarios translated into English).

#### Emotional expressions

Portrayals of shame, sadness, and neutrality by a white man and a white woman were selected from the same UC Davis set of emotional expressions used in Study 1. The pictures were re-sized to fit a 377×457 px space and placed against a black background.

### Measures

After reading the scenario and seeing the portrayal of emotion, participants evaluated the perpetrator on nine-point Likert scales described below, 1 being “Not at all” and 9 being “Very much.” The complete measures are presented in S2 Appendix.

#### Empathy felt towards the perpetrator

Empathy was assessed on a three-item self-report measure developed by Keltner et al. [18], including items such as, “I feel empathy towards the person.” Cronbach’s alpha for the three items was .90, indicating high internal consistency.

#### The perpetrator’s moral sense

Moral sense was measured using four items such as “The person believes that s/he shouldn’t have acted as s/he did” (𝛼 = .78) on a scale adapted from Stearns and Parrott [8].

#### Social anxiety

Social anxiety was measured using three items (𝛼 = .89) also adapted from Stearns and Parrott [8], including statements such as, “The person is concerned that others will disapprove of her/him.”

#### Intention to punish and willingness to cooperate with the perpetrator

No appropriate existing measures were found for cooperation or punishment. The authors, therefore, generated a pool of suitable items and chose four questions with face validity to measure punishment (e.g., “The person should be punished in some way”) and four items to measure cooperation (e.g., “I could imagine myself cooperating with the person”). Both of these measures showed an acceptable level of internal consistency (𝛼 = .79 and .88 for punishment and cooperation, respectively).

### Analysis and design

A 3 (shame vs. sadness vs. neutrality) × 2 (man vs. woman; note that perpetrator gender was only used for counterbalancing purposes and was subsequently collapsed into a single category) repeated-measures experimental design was applied: each participant read six stories during which one of three different emotions was displayed by a man or a woman perpetrator. The order of the expression pictures was counterbalanced. Then, a repeated measures ANOVA model was used to investigate the effect of the emotional expression on five dependent variables: 1) empathy felt towards the transgressor, 2) perceived moral sense of the transgressor, 3) perceived social anxiety felt by the transgressor, 4) intention to punish the transgressor and 5) willingness to cooperate with him/her. Normality of residuals was visually inspected and a Greenhouse-Geisser correction was applied when the sphericity assumption of repeated measures ANOVAs was violated. (Normality plots available in OSF repository: https://doi.org/10.17605/OSF.IO/B5KYM.) When a significant main effect for emotional expression was obtained, a test for within-subject contrasts was carried out.

## Results

### The effect of the emotional expression on empathy, social anxiety, and perceived morality

Repeated measures ANOVAs with the emotional expression (shame vs. sadness vs. neutrality) as a factor were conducted to investigate the effect of the expression on empathy, social-anxiety, and moral sense ratings. The means, standard deviations, and ANOVA statistics regarding each expression category are reported in Table 1. As these show, emotional expression had a significant effect (*p* < .001, η_p_^2^ = .24) on empathy ratings. Contrasts revealed that people reported higher ratings of empathy towards perpetrators expressing shame than neutrality, *F*(1, 59) = 31.33, *p* < .001, *η*_p_^2^ = .35. However, no difference was found between shame and sadness, *F*(1, 59) = 0.07, *p* = .79, *η*_p_^2^ = .001.

**Table 1:** Means and standard errors of empathy, moral judgments, and punitive and cooperative intentions presented as a function of perpetrator’s expression.

Variables	Shame *M (SD)*	Sadness *M (SD)*	Neutral *M (SD)*	Main effect of emotion *F*(2, 118)
Empathy	5.42 (1.63)	5.37 (1.69)	4.16 (1.65)	18.43***†
Moral Sense	6.00 (1.12)	6.27 (1.20)	3.51 (1.26)	101.03***
Social Anxiety	6.72 (1.09)	7.17 (1.31)	4.06 (1.39)	105.16***‡
Cooperation	5.54 (1.54)	5.50 (1.42)	4.82 (1.51)	6.90**
Punishment	4.46 (1.24)	4.35 (1.49)	4.80 (1.63)	2.51

*** p < .001

** p < .01

* p < .05.

†Greenhouse-Geisser F (1.80, 106.25)

‡Greenhouse-Geisser F (1.64, 96.92).

Perpetrators’ emotional expression also affected perceived moral sense (*p* < .001, η_p_^2^ = .63); as hypothesized, those expressing shame after the transgression were perceived as more moral than those expressing neutrality, *F*(1, 59) = 125.10, *p* < .001, *η*_p_^2^ = 0.68. However, no significant difference was found between sadness and shame, *F*(1, 59) = 1.62, *p* = .21, *η*_p_^2^ = .10.

As presented in Table 1, perpetrators’ emotional expression significantly affected perceived social anxiety (*p* < .001, *η*_p_^2^ = 0.64). Contrasts revealed that perpetrators expressing sadness were perceived as more socially anxious than those expressing shame, *F*(1, 59) = 6.60, *p* = .013, *η*_p_^2^ = .10, or neutrality, *F*(1, 59) = 95.69, *p* < .001, *η*_p_^2^ = .62 (for the mean values, see Table 1).

Taken together, our hypothesis (H3) that displaying shame after norm violation would produce higher ratings of empathy, moral sense, and social anxiety as compared expressions of sadness or neutrality did not get clear support. While the difference between nonverbal shame and neutral expression was in alignment with the hypothesis, there were no differences in social appraisals between the displays of shame and sadness.

### The effect of the emotional expression on cooperation and punishment

Equivalent ANOVAs were conducted separately for the cooperation and punishment ratings. Contrary to assumptions, emotional expression did not predict punitive intentions, *p* = .09 (see Table 1).

As predicted, perpetrators’ emotional expression significantly influenced the participants’ cooperative intentions, *p* = .001, η_p_^2^ = .11. Within-subject contrasts of cooperation ratings supported the pattern reported in Table 1, in that people were more willing to cooperate with those who expressed shame after a norm violation than those showing a neutral face, *F*(1, 59) = 9.47, *p* = .003, *η*_p_^2^ = .14. Again, no difference was found between shame and sadness, *F*(1, 59) = 0.03, *p* = .87, *η*_p_^2^ < .001. Consequently, no support was received for the hypothesis (H4) that displaying shame would lead to decreased punitive intentions and increased cooperative intentions as compared to displays of sadness, while an expected difference was observed between shame and a neutral expression.

## Summary study 2

Perpetrators’ expressions of shame and sadness produced higher ratings of empathy, social anxiety, moral sense and a willingness to cooperate than the expression of neutrality. Perpetrators expressing sadness after a norm violation were perceived as slightly more socially anxious when compared to those expressing shame but no noticeable differences appeared between perpetrators expressing shame and sadness in terms of other outcomes. Contrary to our hypothesis, perpetrators’ shame (or sadness) displays did not lower observers’ inclination to punish the perpetrator.

## Study 3

### Aims

Several changes were made to the experimental set-up to carefully scrutinize possible explanations for the unexpected results obtained in Study 2 and to replicate the obtained findings. A between-subjects design was adopted to better ensure the naïveté of participants and to see whether the effects of perpetrators’ emotional expressions were different if the perceiver was exposed to only one transgression scenario. Indeed, in everyday life, people rarely encounter a series of events in which another person violates moral standards and sequentially responds to one’s wrongdoing in differing ways.

Moreover, given that expressions of shame are particularly effective in changing the perceiver’s social intentions if expressed after more serious norm transgressions [18], we examined also whether the social consequences of displaying shame depended on transgression severity. Specifically, we hypothesized (H5) that there would be no difference in the inclination to punish perpetrators of milder transgressions depending on their emotional expression; but after a more serious transgression, expressing shame as compared to other emotions would result in reduced inclination to punish the perpetrator. To test this hypothesis, the effect of nonverbal displays of shame on intentions to punish a perpetrator was contrasted to situations in which the transgressor was expressing sadness, anger, or neutrality after getting caught violating a moral norm. We assumed that reacting to the situation with an expression of anger would be observed as hostility and open rebellion of the established moral code. As a stark contrast to submission and conformity conveyed by nonverbal shame, expressions of anger were thought to result in stronger intentions to punish and attenuated cooperative intentions as compared to shame displays. The addition of an angry-expression condition was further motivated by the finding in Study 1 that a perpetrator expressing anger was rated as more dominant and more negatively valenced of all of the moral appraisals as compared to a person expressing shame, sadness or neutrality.

Finally, Study 3 aimed also to investigate the mediating role of empathy, moral sense, and social anxiety in the relationship between perpetrators’ emotional expressions and observers’ punishment and cooperation intentions. Based on the findings of Study 2 and earlier studies [8, 18], it was hypothesized (H6) that shame displays would evoke empathy in the perceiver and increase the perceived moral sense and social anxiety of the perpetrator, which in turn would promote cooperative intentions and buffer against intentions to punish the perpetrator. In other words, the test was simply whether empathy, social anxiety, and/or moral sense would mediate effects of nonverbal shame on cooperative and and/or punitive intentions but no specific assumptions were made about the mediators’ relations to each other (i.e. the mediators were entered into the model as competing factors).

## Method

### Participants

The recruitment was done through student organization mailing lists at the University of Helsinki. A sample of 924 participants with *M*_age_  = 29.9, *SD* = 9.96 (73.7% women) decided to take part in this online experiment, which participants accessed through a link posted in the advertisement email. There were no restrictions for participation and all willing participants were included in the sample. Participants were told that their responses would be fully anonymized and that they would have the right to withdraw from the study at any time in which case their data would be removed. *PANGEA* software [24] was used to determine that 924 participants would grant 80% power to detect Cohen’s *d* = 0.26 size effect for emotion expression × severity of transgression interaction when a 4 × 2 between-subject design and an alpha level of .05 were applied.

### Transgression scenarios

A new set of transgression scenarios was created where the harmful implications of the transgressions were either the same as in Study 2 or more severe. Specifically, three scenarios from Study 2 were selected (see S1 Appendix scenarios 1, 2, and 6) to represent the less-severe transgressions and then, based on the same scenarios, altered versions were created in which the harmful implications of the transgressions were amplified by increasing the amount of material damage or physical injury described. A sample scenario is presented below (alteration in comparison to the less severe version is bolded):

“A young man is standing on a subway platform waiting for a train, which is running late. The platform is crowded with people. When the train finally arrives, the man makes a dash for the carriage. However, it is difficult to move in the crowd and people are jostling each other. This irritates the man, who tries to push the people in front of him even harder, even though there is no room for them to move. The pushing makes the person in front of the man fall over and **get severely injured**. Other passengers are looking disapprovingly at him.”

### Procedure

Before the presentation of scenarios and pictures, participants indicated their gender and age. In each trial, a written moral transgression scenario (more or less severe) was presented with either a man or woman protagonist. After reading the scenario, participants were asked to press the “enter” key on their keyboard to see a photograph of the protagonist expressing one of four emotions (shame, anger, sadness, or neutral) with their face and body posture after getting caught. (Expression pictures were selected from the same set that was used as in Study 1 & 2; approx. 377×457 px each.) The picture was presented for ten seconds after which the participants were asked to fill out questionnaires about 1) empathy felt towards the transgressor, 2) perceived moral sense of the transgressor, 3) perceived social anxiety felt by the transgressor, 4) intention to punish the transgressor and 5) willingness to cooperate with him/her (i.e., the same measures used in Study 2), in the respective order. The whole procedure was presented online through www.psytoolkit.org and took five to ten minutes to complete.

### Design and analyses

A 3 (scenarios) x 2 (severity of transgression: more vs less severe) x 2 (man vs woman) x 4 (emotional expression) between-subjects design was implemented, with every participant seeing one of the 48 possible combinations. The effects of the gender of a perpetrator and/or specific scenarios were not topical for the current study and were thus controlled in the study design. The data was accordingly aggregated before the analysis, leaving a 2 (severity of transgression) x 4 (emotional expression) design. To test for effects of emotions and severity of the scenarios, we computed univariate ANOVAs on the five dependent variables as well as planned contrasts between shame and the other three emotional expressions. Before conducting the ANOVA, normality of residuals was visually inspected and assumption of homogeneity of variance between the comparison groups was confirmed with Levene’s test (all *p*s higher than .05; see S3 Appendix for the complete results; normality plots available in OSF repository: https://doi.org/10.17605/OSF.IO/B5KYM).

Finally, to see if empathy, moral sense and/or social anxiety would mediate the emotion expressions’ effect on cooperation and/or punishment, a parallel multiple mediator analysis was conducted in which each mediator’s unique contribution for the dependent variable was evaluated within the same model (see [31]). PROCESS 3.1 macro [32] was used for IBM SPSS 24, which is an OLS regression-based path analysis approach to mediation. PROCESS model 4 was selected where emotional expression was a categorical independent variable, empathy, moral sense, and social anxiety were mediators, cooperation and punishment were dependent variables, and severity of transgression a covariate. Because the effect of shame was the main interest of the study, shame was used as a contrasting category for emotion expression. Relative direct and indirect effects 95% CI’s and SE’s were estimated from 5000 percentile bootstrap samples.

## Results

### The effect of the emotional expression on empathy, social anxiety and perceived moral sense, and interaction effects with the severity of the transgression

For the empathy outcome variable, the ANOVA revealed significant main effects of emotional expression, *F*(3, 916) = 14.61, *p* < .001, *η*_p_^2^ = .05, and severity of the transgression, *F*(1, 916) = 47.59, *p* < .001, *η*_p_^2^ = .05, with the more severe transgression leading to lower levels of empathy (*M*_less severe transgression_ = 4.74, *SD* = 1.96; *M*_more severe transgression_ = 3.90, *SD* = 1.92;). However, no interaction effect between these two factors was found, *F*(3, 916) = 1.34, *p* = .26, *η*_p_^2^ = .004. Pairwise comparisons showed that levels of empathy did not differ between shame display (*M* = 4.80, *SD* = 2.00) and expression of sadness (*M* = 4.77, *SD* = 1.97; *p* = .46, *η*_p_^2^ = .001) but did differ between shame and angry expression (*M* = 4.07, *SD* = 1.90; *p* < .001, *η*_p_^2^ = .02) and between shame and neutral expression (*M* = 3.88, *SD* = 1.93; *p* < .001, *η*_p_^2^ = .03).

Judgments of perpetrator’s moral sense differed by expressed emotion, *F*(3, 916) = 94.25, *p* < .001, *η*_p_^2^ = .24), but not by transgression severity, *F*(3, 916) = 2.97, *p* = .09, *η*_p_^2^ = .003, and there was no significant interaction effect between the two factors, *F*(3, 916) = 0.46, *p* = .71, *η*_p_^2^ = .002. The pairwise comparisons between shame display (*M* = 5.83, *SD* = 1.60) and the other expressions revealed differences between shame and all other emotions (sadness: *M* = 5.48, *SD* = 1.61; *p* = .017, *η*_p_^2^ = .01; anger: *M* = 3.96, *SD* = 1.58; *p* < .001, *η*_p_^2^ = .15; neutral: *M* = 3.84, *SD* = 1.66; *p* < .001, *η*_p_^2^ = .18).

Perpetrator’s perceived social anxiety was also influenced by the expressed emotion, *F*(3, 916) = 97.85, *p* < .001, *η*_p_^2^ = .243), but not by transgression severity, *F*(3, 916) = 3.06, *p* = .08, *η*_p_^2^ = .003, and again no significant interaction effect was found between the two, *F*(3, 916) = 0.03, *p* = .99, *η*_p_^2^ < .001. Shame display (*M* = 6.49, *SD* = 1.73) differed significantly from angry (*M* = 4.92, *SD* = 1.83; *p* < .001, *η*_p_^2^ = .10) and neutral expression (*M* = 4.17, *SD* = 1.74; *p* < .001, *η*_p_^2^ = .189) but not from sad expression (*M* = 6.39, *SD* = 1.59; *p* = .46, *η*_p_^2^ = .001).

### The effect of the emotional expression on cooperation and punishment, and interaction effects with severity of transgression

Regarding observers’ intention to punish the perpetrator, we found a significant main effect only for severity of transgression, *F*(1, 916) = 76.63, *p* < .001, *η*_p_^2^ = .08, with the expected effect direction (*M*_less severe transgression_ = 4.37, *SD* = 1.64; *M*_more severe transgression_ = 5.35, *SD* = 1.80). However, neither the main effect of emotional expression, *F*(3, 916) = 2.07, *p* = .10, *η*_p_^2^ = .01, nor the interaction between the severity and emotion expression, *F*(3, 916) = 0.12, *p* = .95, *η*_p_^2^ < .001, were significant. Therefore, no support was found for H5, which predicted that, after a more severe transgression, expressing shame as compared to other emotions would result in reduced inclination to punish the perpetrator.

Intention to cooperate with the perpetrator showed significant main effects of emotional expression, *F*(1, 916) = 13.34, *p* < .001, *η*_p_^2^ = .04, transgression severity, *F*(1, 916) = 25.16, *p* < .001, *η*_p_^2^ = 0.03), and their non-significant interaction effect, *F*(3, 916) = 1.03, *p* = .38, *η*_p_^2^ = .003. The participants were less willing to cooperate with the perpetrator committing a more severe (*M* = 4.23, *SD* = 1.99) than less severe transgression (*M* = 4.88, *SD* = 1.91). However, if the perpetrator expressed sadness or shame after the norm violation, observers’ (i.e., participants’) willingness to cooperate remained equally high (shame: *M* = 5.11, *SD* = 1.94; sad: *M* = 4.89, *SD* = 1.91; *p* = .08, *η*_p_^2^ = .003), and significantly higher than when the perpetrator expressed anger (*M* = 4.28, *SD* = 1.96, *p* < .001, *η*_p_^2^ = .03) or neutral emotional state (*M* = 4.20, *SD* = 1.91, p < .001, *η*_p_^2^ = .03). The observation replicated and extended the findings of Study 2 by showing also the effect of expressing anger in the context.

### Indirect effects of emotional expression via empathy, moral sense, and social anxiety on cooperation and punishment

**Fig 5:**
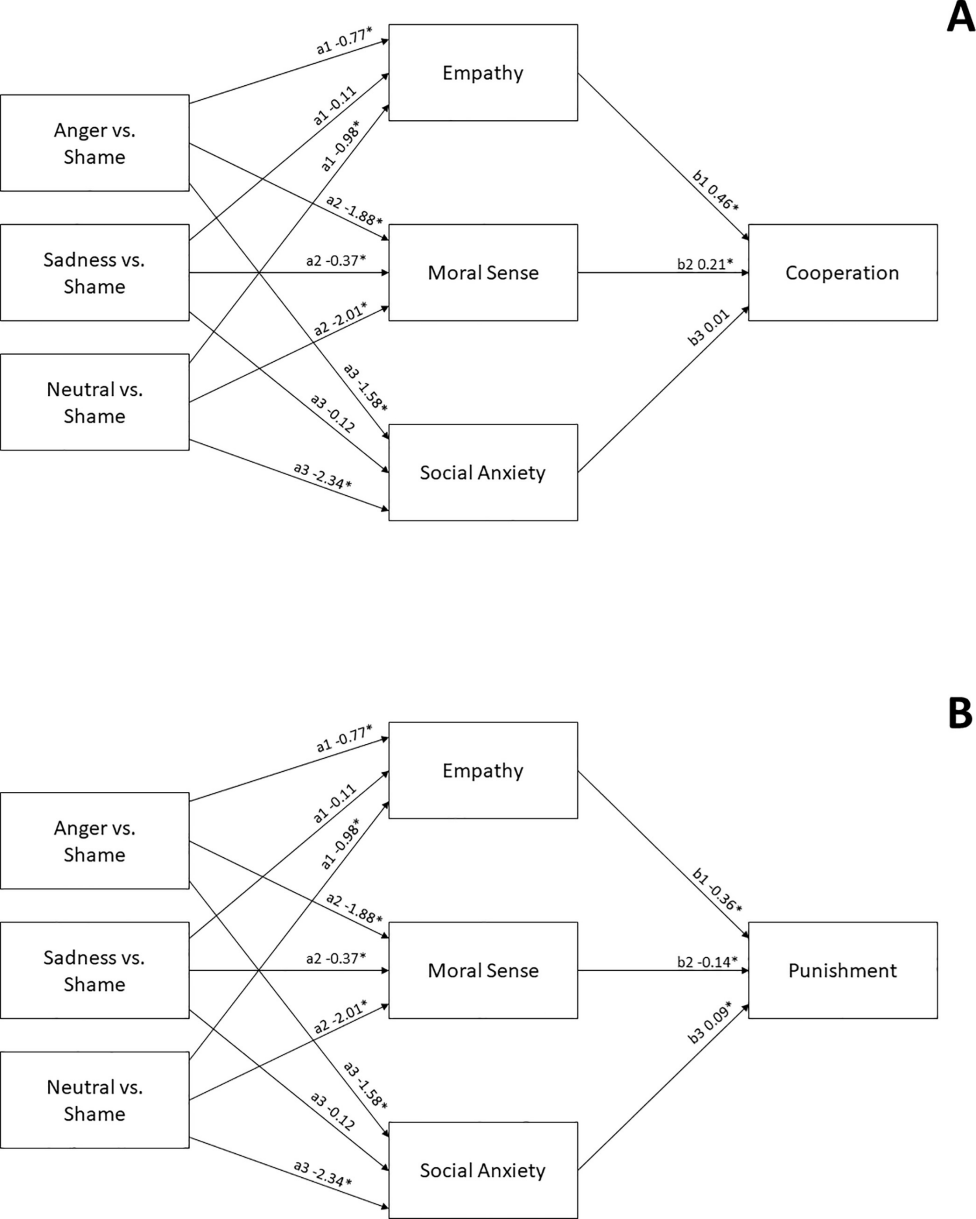
Regression model of emotional expressions’ effect on mediators and mediators’ relation to cooperation and punishment ratings. Coefficients are unstandardized regression coefficients, emotional expressions were contrasted with shame when comparing independent to mediator, and severity of transgression was used as a covariate. A’s refer to emotional expressions' influence on mediators and b’s to mediators’ effect on cooperation and punishment when all the other mediators in the model are controlled for. For the complete output of the regression results see S4 Appendix.

To test whether (H6) empathy, moral sense, or social anxiety mediated the influence of emotional expression on punishment and cooperation, relative direct and indirect effects (see Table 2) and direction (±) of the relation between emotional expression to mediators (*a*) and mediators to punishment and cooperation (*b*) were tested (see Fig 5), following the analysis protocol of Hayes [32]. Since high levels of multicollinearity can be a problem for regression-based methods, correlations between mediators, tolerance values and variance inflation factors were inspected and deemed acceptable before performing the mediation analysis (see S3 Appendix).

**Table 2:** Relative direct and indirect effects of emotional expressions contrasted to shame on cooperation and punishment estimated with ordinary least squares path analysis.

	Y: Cooperation				Y: Punishment			
	effect	bSE	bLLCI	bULCI	effect	bSE	bLLCI	bULCI
**Shame vs Anger**								
Relative direct effect (c’)	-0.10	0.16	-0.41	0.22	-0.06	0.16	-0.36	0.25
*Relative indirect effects (ab)*								
expression→empathy→Y	-0.35*	0.07	-0.53	-0.19	0.28*	0.07	0.15	0.42
expression→moral sense→Y	-0.39*	0.09	-0.58	-0.22	0.27*	0.09	0.10	0.44
expression→social anxiety→Y	-0.02	0.07	-0.15	0.11	-0.14*	0.06	-0.26	-0.02
**Shame vs Sadness**								
Relative direct effect (c’)	-0.16	0.15	-0.46	0.13	0.05	0.15	-0.23	0.33
*Relative indirect effects (ab)*								
expression→empathy→Y	-0.05	0.09	-0.22	0.12	0.04	0.07	-0.09	0.17
expression→moral sense→Y	-0.08*	0.04	-0.16	-0.01	0.05*	0.03	0.01	0.11
expression→social anxiety→Y	-0.002	0.01	-0.02	0.02	-0.01	0.02	-0.04	0.02
**Shame vs Neutral**								
Relative direct effect (c’)	-0.06	0.17	-0.38	0.28	-0.14	0.16	-0.46	0.19
*Relative indirect effects (ab)*								
expression→empathy→Y	-0.45*	0.09	-0.63	-0.28	0.36*	0.07	0.22	0.51
expression→moral sense→Y	-0.42*	0.10	-0.62	-0.23	0.29*	0.09	0.11	0.46
expression→social anxiety→Y	-0.03	0.10	-0.23	0.16	-0.21*	0.09	-0.38	-0.03

Effects are unstandardized regression coefficients, and bSE, bLLCI, and bULCI refer to percentile bootstrapped (5000 samples) standard error, and lower and upper 95% confidence intervals, respectively. The effect of emotional expression is always contrasted to shame. Y is short for the dependent variable. The severity of transgression was used as a covariate. All mediators were included in the model at the same time. For complete output of the regression results see S4 Appendix.

* = CI does not contain 0.

Fig 5A shows that, relative to shame display, seeing a perpetrator expressing anger resulted in decreased empathy (a1 = -0.77, p < .05, Fig 5A) toward the perpetrator. Empathy, in turn, was positively associated with intention to cooperate with the perpetrator (b1 = 0.41, p < .05, Fig 5A), which translated into an indirect effect of perpetrators’ emotional expression to observers’ cooperative intentions via empathy felt towards the perpetrator (ab = -0.35, *p* < .05, see the left side of Table 2), which lends support to H6. Expressing anger instead of shame also led participants to rate perpetrators’ moral sense less favorably (a2 = -1.88, *p* < .05). High moral sense, in turn, predicted increased intention to cooperate with the perpetrator (b2 = 0.21, *p* < .05), this entire pattern translating into an indirect effect of perpetrators’ emotional expression to observers’ cooperative intentions via perceived moral sense (ab = -0.39, *p* < .05, see the left side of Table 2) that was also in line with H6. Perpetrators expressing anger instead of shame were also perceived as less socially anxious (a3 = -1.58, *p* < .05). However, social anxiety had no significant association with cooperative intentions (b3 = 0.01, *p* > .05), giving no support for H6 in regard to social anxiety. Taken together, these results suggest that expressing anger as compared to shame results in reduced empathy and perceived moral sense in the observers, which in turn reduces their willingness to cooperate with the perpetrator.

When seeing a perpetrator express sadness instead of shame, participants felt as much empathy towards him/her but rated the perpetrator’s moral sense lower (a2 = -0.37, p < .05, Fig 5 and 5A). Since perception of moral sense increased cooperation, this led to a small indirect effect (ab = -0.08, *p* < .05, see the left side of Table 2), indicating that expressing shame instead of sadness after a norm violation led observers to evaluate the perpetrator’s moral sense more favorably, which in turn increased their willingness to cooperate with the transgressor. Again, social anxiety was not found to mediate the relationship between expression and cooperation. Then, seeing the perpetrator in a neutral state instead of displaying shame following a norm violation led the participants to feel less empathy (a1 = -0.98, *p* < .05, Fig 5A), and evaluate the perpetrator’s moral sense less favorably (a2 = -2.01, *p* < .05, Fig 5A). Since both empathy and perceived moral sense had positive associations with cooperative intentions, this led to an indirect effect in which the perpetrator’s neutral expression after a violation decreased observers’ cooperative intentions via reduced empathy and perceived moral sense (ab = -0.45 and ab = -0.42, respectively).

Fig 5B presents the regression coefficients between emotional expressions, mediators, and punitive intentions. As can be seen, higher empathy ratings predicted significantly lower punitive intentions (b1 = -0.36, *p* < .05). Given that seeing the perpetrator express anger instead of shame decreased observers’ empathy, this pattern translated into a significant indirect effect of emotion expression on punitive intentions via empathy (ab = 0.28, *p* < .05, see Table 2, right side). A similar indirect effect on punitive intentions via empathy was also observed when contrasting shame display to neutral expressions (ab = 0.36, *p* < .05, see Table 2, right side), but not when contrasting shame to sadness (ab = 0.04, *p* < .05). In other words, the perpetrator's expression of shame and sadness resulted in higher empathy in the observers, which, in turn, resulted in their decreased willingness to punish the perpetrator. Support for H6 was thus observed in terms of empathy.

From Fig 5B, one can also see a significant negative association between perceived moral sense and punitive intentions (b2 = -0.14, p < .05). Since displaying shame instead of anger, neutrality or sadness made observers perceive the perpetrators’ moral sense more favorably, the pattern indicated an indirect effect of shame display on punitive intentions that was mediated by perpetrators’ perceived moral sense. Indeed, the relative indirect effects of emotional expressions on punitive intentions via moral sense were all statistically significant, also when contrasting shame to sadness (ab = 0.05, *p* < .05, see Table 2, right side), giving clear support for H6.

Finally, when evaluating the mediating role of a perpetrator’s perceived social anxiety on punitive intentions, an unexpected, small positive association was found between the mediator and the outcome (b3 = 0.09, *p* < .05). Given that angry expression was considered as less anxious compared to shame display (a3 = -1.58, *p* < .05), the significant relative indirect effect (ab = -0.14, *p* > .05) indicated that expressing anger instead of shame after violating a norm resulted in decreased punitive intentions. A similarly counter-intuitive indirect appeasement effect was found when contrasting shame to neutral expression (ab = -0.21, *p* > .05). To further investigate these surprising findings, separate mediation models were calculated for each individual mediator. Individual models revealed that when empathy and moral sense evaluations were not controlled for, increase in social anxiety did, as expected, lead to a decrease in punishment (b = -.12, *p* < .05) and this translated into an indirect appeasement effect for shame compared to neutral and anger expression (ab = 0.28 and 0.19, *p*’s < .05, respectively) in accordance with H6. Also, the effect of emotional expressions on cooperation via social anxiety became significant when empathy and moral sense were not included in the model. The indirect effects via empathy and moral sense remained the same in terms of direction and magnitude when the mediators were included individually to the model or included in the same model (for complete output of these individual mediation models see S4 Appendix).

Follow-up analyses suggested that the previously found positive relation between social anxiety and punitive intentions was a result of intercorrelations between the mediators. To make sure this was the case, we concluded the analysis calculating partial correlations of the social anxiety, empathy, moral sense, and punishment ratings. The correlation between social anxiety and punishment was *r* = -.14, *p* < .001, but when moral sense or empathy ratings were controlled for, the direction of the relationship changed (*r* = .06, *p* = .09 and *r* = .04, *p* = .26, respectively), even more prominently if both variables (moral sense and empathy) were controlled for (*r* = .09, *p* = .006). Likewise, the relationship between cooperation and social anxiety collapsed when the partial correlation controlling for empathy and moral sense ratings was calculated (from *r* = .32, *p* < .001 to *r* = .01, *p* = .74). Altogether, these analyses confirmed that the appeasement and cooperative qualities of social anxiety were a result of intercorrelations between social anxiety and empathy/moral sense evaluations and that social anxiety in itself did not have a significant contribution as a mediator in this context.

Finally, as can be seen from Table 2, none of the relative direct effects of emotional expression were significant, suggesting that when the effects of mediators were controlled for, none of the expressions contrasted to shame had a direct effect on cooperation or punishment. Using Baron and Kenny’s [33] terminology, this indicates that the mediation effects described above represent complete rather than partial mediation (i.e., when the model is adjusted for the effect of the mediator, the main effect of the predictor not only weakens, but becomes statistically non-significant and drops close to zero, which implies that the effect of the predictor to the dependent variable is conveyed completely through the mediator).

## Summary study 3

Perpetrators displaying shame or sadness were seen as more socially anxious and having higher moral sense than perpetrators expressing neutrality or anger after a transgression of social norms. Expressions of shame and sadness also increased observers’ empathy and willingness to cooperate with the perpetrator. While transgression severity also influenced the empathy felt toward the perpetrator, and observers’ intention to punish and cooperate with the perpetrator, there was no interaction between the emotional expression of a perpetrator and severity of the norm violation. Interestingly, and similar to Study 2, perpetrators’ emotional expression did not influence observers’ punitive intentions. However, mediation models revealed that the appeasement effect of emotional expression did emerge via more positive evaluations of perpetrators’ moral sense and the empathy felt towards them. In other words, the participants who perceived perpetrators’ moral sense more favorably, or felt more empathy due to the perpetrators’ expression of shame or sadness, were less willing to punish the perpetrator. This indirect appeasing effect of shame display via moral sense was found to be even stronger than the indirect effect of expression of sadness to punitive intentions. Indirect effects of shame display via moral sense to cooperative intentions were similarly stronger than the indirect effects of sad, angry or neutral expressions to cooperative intentions. While perpetrators’ perceived social anxiety initially seemed to increase rather than decrease the observers’ punitive interventions, this relationship was further found to be a result of strong intercorrelations between social anxiety and other mediators.

## Discussion

The current research examined the social functions of nonverbal displays of shame in the context of everyday norm violations. Previous theoretical considerations suggest that displaying shame through slumped posture and averted gaze appeases observers and informs them of the perpetrator’s moral sense and cooperative reliability [11, 17]. Thus far, a relatively limited amount of experimental evidence had been put forward to demonstrate the appeasing social function of nonverbal shame display. In fact, much of the empirical support for this function had been obtained by examining observers' reactions to rule breakers’ verbal expressions of shame [8–11]. The present study strived to close this knowledge gap by experimentally testing effects of nonverbal shame display on observers’ moral decision-making and investigating the appraisal mechanisms that drive such effects.

In Study 1, we found that the display of shame was perceived similarly to the nonverbal expression of sadness in terms of affective valence, arousal, and dominance ratings, but better communicated the perpetrators’ understanding of their wrongdoing than expressions of sadness or any other emotion. Shame displays were also seen as the most socially appropriate reaction to one’s norm violation, although the expression of sadness was also considered appropriate. Altogether, the results of Study 1 gave support to the functional account that nonverbally displayed shame is an optimal way to convey one’s renewed conformity to established moral standards [11, 17].

In Study 2, participants were presented with a hypothetical situation in which a person had violated the established moral norms and afterward displayed nonverbal shame, sadness, or a neutral emotional state. Perpetrators displaying nonverbal shame were evaluated as more anxious and having a higher moral sense than perpetrators who had a neutral expression. The findings were in line with previous research showing that expressing shame verbally after wrongdoing elicited more positive evaluations of the perpetrator’s moral sense [8, 9]. Also in line with previous work [18], we found that perpetrators displaying shame evoked more empathy in observers than those expressing neutrality after their violation. However, contrary to our assumption of the uniqueness of shame display, the effects of sadness were comparable to those of shame and there were no differences in the social effectiveness of the two displays in any of the above-mentioned social appraisals. Likewise, displays of shame and sadness were equally effective in increasing observers’ willingness to cooperate with the perpetrator when compared to a neutral expression. Finally, we found that neither shame nor sadness diminished observers’ willingness to punish the perpetrator. These unexpected findings lead us to conduct a follow-up experiment with a large sample size and a modified experimental design.

In this experiment (i.e., Study 3), participants encountered a hypothetical scenario in which the protagonist first violated a moral norm and then displayed nonverbal shame, sadness, anger, or a neutral emotional state via bodily expressions. The violation severity was varied between mild and moderate to find out if the social function of displaying shame was influenced by violation severity. The key findings of the previous study were replicated. For example, displays of shame and sadness both increased perceived social anxiety, empathy, and willingness to cooperate with the perpetrator as compared to angry and neutral expressions. Similar to Study 2, observers’ willingness to punish the perpetrator was not influenced by the emotional expression. Unlike Study 2, however, the moral sense of perpetrators displaying shame was evaluated more positively than perpetrators expressing sadness. Interestingly, we found that the influence of emotional expression was not dependent on transgression severity.

When examining whether appraisals of social anxiety, moral sense and empathy mediated the effects of emotional expressions on cooperative and punitive intentions, both empathy and moral sense were found to mediate the link between perpetrators’ expressions and observers’ behavioral intentions. The effects of expressed shame and sadness on punitive and cooperative intentions were similarly mediated by observers’ empathy towards the perpetrator. However, the link from expressions via moral sense to punitive and cooperative intentions was stronger in the context of shame than sadness. What came to the perceived social anxiety, its mediating effect was less clear: initially, increase in social anxiety was found to lead to a harsher punishment and having no effect on cooperation. However, if mediators were added to the statistical model separately, social anxiety decreased willingness to punish the perpetrator and increased the cooperative intentions, as hypothesized. Statistically speaking, social anxiety’s effect on appeasement and cooperation seems to be largely accounted for by moral sense and empathy ratings. On the conceptual level, however, this finding could hint that social anxiety, moral sense and empathy are parts of the same, possibly sequential, appraisal process. The perceiver could first see the perpetrator in an anxious state, which would then inform the perceiver about the perpetrator’s understanding of his/her wrongdoing and evoke feelings of empathy in the perceiver. Social anxiety could thus work as a distal factor in the appraisal process while empathy and moral sense evaluations would more directly regulate the perceiver’s behavioral intentions.

Below we will reflect on what the other findings of Studies 1–3 tell about the functionality of expressing shame in the context of norm violations. In this elaboration, we will focus on two critical aspects of the functional account to nonverbal shame: the appeasement assumption and the uniqueness assumption.

### Revisiting the appeasement assumption of nonverbal shame display

What comes to the appeasement assumption, there are strong theoretical reasons and circumstantial empirical evidence supporting the appeasing influence of shame displays. However, careful screening of the literature reveals that few studies have actually demonstrated a direct effect of shame displays on reduced punitive behaviors. Much of the early evidence on the appeasement effects of nonverbal shame comes from a review by Keltner, Young, and Buswell [18]. In their review, the case was based on an unpublished mock trial study, which showed that a defendant showing shame received a shorter sentence than a defendant showing a neutral expression or an expression of contempt. However, the fact that the study was unpublished makes it difficult to evaluate the results’ validity. In fact, when published findings are considered, the link between nonverbal shame displays and appeasement is less clear. For example, Keltner et al. [18] reported that shame displays produced stronger empathy in observers, which was interpreted as support for the appeasement hypothesis. However, in this case the inclination to punish the perpetrator was not measured. Then, Proeve and Howells [9] found that verbal expressions of shame in a trial context led to a more favorable evaluation of the person, but no difference was observed when it came to sentencing. Recently, Hornsey et al. [19] examined the effects of “embodied remorse” (which was operationalized as either kneeling or crying) in the context of making an apology and found out that even though crying and kneeling did lead to more overall positive evaluations of the person, these nonverbal cues did not noticeably increase observers’ willingness to forgive. Thus, it is not clear whether displaying shame with either words or body language actually translates to a lighter punishment.

Similar to the aforementioned work, we did not find that perpetrators' shame displays reduced observers’ punitive intentions in Study 2 or 3. However, when the indirect link between shame display and punitive intentions was investigated, perceptions of perpetrator’s moral sense and the empathy felt toward the perpetrator were found to result in decreased willingness to punish. That is, showing shame after a norm transgression seemed to elevate participants’ evaluation of the perpetrator’s moral sense and increase the empathy felt toward them (compared to other expressions); and subsequently, those participants who felt more empathy and/or had higher opinions of the perpetrator’s moral sense were less willing to punish the perpetrator.

Although modern literature on methodology agrees that there does not need to be a total effect (i.e., a main effect) in order to find an indirect effect in mediation analysis (for example [32, 34, 35]), it is sensible to consider some possible explanations for why only an indirect effect between shame display and punitive intentions was found. The literature listed above suggests a multitude of methodological reasons for this pattern, including small sample sizes, variance in measurement precision between variables, and contradicting effects in different subpopulations. Since most of the strictly methodological reasons (e.g., small sample size) are implausible in the case of Study 3, we will focus on explanations that are more closely related to psychological and situational factors related to the experiment itself.

One possible explanation is related to the perceived costs of punishing the transgressor. Since in a hypothetical situation punishing the transgressor does not impose any real costs on the participant, and given that people are generally motivated to punish rule breakers [36], it is possible that subtle social cues like the perpetrator’s emotional expressions are ineffective in reducing the observers’ motivation to punish. In fact, in Study 3 the transgression severity influenced the observer’s punitive intentions while the emotional expression did not. It is also possible that displaying shame works as an appeasement device only in very limited circumstances, say, for example, when explicitly violating an established dominance hierarchy (e.g., [17]).

Yet another possibility is that the submissive qualities of shame influence others’ behaviors only after the transgressor has been punished, and the community must then decide whether to exclude the transgressor from the group. A person who accepts the group’s judgment and the punishment that follows without defiance might be perceived as a more attractive member than one who openly rebels against the group and/or shows indifference to the negative assessments. Our consistent finding, that displaying shame increased the observers’ willingness to cooperate while not saving the perpetrator from punishment, supports this interpretation. The finding that displaying shame after a norm violation promotes cooperative intentions in the observers is also fully in line with Fessler’s functional account [17]. However, future studies should investigate more closely the possible situational factors that modulate the social functions of shame, examining also the possible post-punishment benefits that the display evokes.

### The unique social function of nonverbal shame displays

Another critical aspect of the functional account that we want to revisit is the assumption of uniqueness. Our three studies gave systematic support for the Fessler’s [17] notion that nonverbal display of shame is exceptionally effective in conveying an understanding of the moral standards and readiness to conform to them. However, we round this quality was not exclusively limited to shame display, but was also associated with sadness. While in Study 1, displays of shame was perceived to better communicate the transgressor’s willingness to accept the consequences of his/her actions than expression of sadness (or any other expression), in Study 2 shame did not differ from sadness in its effects on any of the social appraisals or behavioral intentions. In Study 3, perpetrators’ moral sense was interpreted more favorably if the perpetrator reacted with shame than with a sad expression, but there were no differences between the two displays in relation to empathy, perceptions of social anxiety, or in relation to punitive or cooperative intentions.

Therefore, these results indicate that nonverbal displays of shame and sadness share some social qualities and influence the recipient in very similar ways when expressed in the context of a norm violation. These common qualities may, for example, be related to submissiveness and negative valence ([37]; see also Study 1 results concerning the affective dimension). Moreover, both emotions are relatively intense, which could convince observers that the perpetrator is taking the situation seriously. In fact, according to anthropological field reports, nonverbal expressions of sadness are often used to appease the recipient. Boehm [13], for example, described incidents among tribe members in which individuals used crying to appease observers after being caught cheating during food acquisition. Similarly, studies conducted on Western children show that both shame and sadness are oftentimes displayed simultaneously in contexts of failure [38].

In favor of the uniqueness assumption, however, Study 3 demonstrated that displaying shame led to overall more favorable views of the perpetrator’s moral sense, which in turn predicted lower risks for punishment and social exclusion. One possible explanation for this finding is that expressing shame better convinces observers that the transgressor is truly feeling remorse about the rule violation and is not just generally distressed about others’ disapproval, thus indicating that the transgressor better understands and accepts the community’s social norms. Support for this claim was also found in Study 1, in which the display of shame was seen as a more appropriate reaction after norm violation, and to better communicate that the transgressor was willing to accept the consequences of his/her actions when compared to the expression of sadness. Therefore, results suggest that shame might have a special role in convincing the observers that the perpetrator’s moral compass is functioning properly, although having otherwise-similar implications as the expression of sadness for observers’ behavioral intentions and feelings of empathy.

### Limitations and conclusions

While we can conclude that displaying shame after norm violation leads to overall more positive evaluations anyone violating social norms and prevents the social exclusion of that person, more research is needed to better understand the situational and person-level factors that moderate the link between shame displays and observers’ punitive intentions. At this point, we can conclude that the appeasing effect of shame is more complex than previously thought and seems to be conditional on observers’ social appraisals and feelings of empathy towards the perpetrator. When it comes to generalizability of these results, there are a number of limitations that should be made clear. Notably, our samples consist of Western university students, which limits examination of cross-cultural differences and similarities [39]. Also, the current results were obtained using a single set of posed emotional expression pictures. In the future, pictures and videos of spontaneous shame displays should be used to further investigate displays’ social functions. Finally, it would be advisable to replicate the current findings with triggers other than vignette paradigms: for instance, using social decision-making paradigms. Despite these limitations, however, the research presented here makes a clear contribution to the literature, as it consistently demonstrates the social functions of nonverbal shame in the context of norm violations and demonstrates how social appraisals of a perpetrator’s moral sense feelings of empathy directed toward the perpetrator drive these functions. Moreover, the research reported here raises new questions about the assumption of expression-specific effects on observers’ moral judgments, revealing the complementary influence of different bodily emotion cues on social decision-making.

## Supporting information

S1 Appendix Scenarios used in Study 2 and 3. (DOCX)

S2 Appendix Scales used in Study 1, 2, and 3. (DOCX)

S3 Appendix Multicollinearity and homogeneity diagnostics for Study 3. (DOCX)

S4 Appendix Individual and parallel mediation models for Study 3. (DOCX)
