# Supplementary material for: How bodily expressions of emotion after norm violation influence perceivers’ moral judgments and prevent social exclusion: A socio-functional approach to nonverbal shame display
Source: PLoS One. 2020 Apr 30;15(4):e0232298. doi: 10.1371/journal.pone.0232298 (PMC7192454; doi:10.1371/journal.pone.0232298)
Supplement: S1 Appendix — (DOCX) [file pone.0232298.s001.docx]

**Study 2**

**S1**. A young woman/man arrives at the front door of her/his apartment building late in the evening. The woman/man has a key to the door but it does not open right away. This makes the woman/man angry and she/he starts to pull the door handle with both hands and finally kicks it. The door opens but the pulling and the kick have broken the lock. In addition, the noise has awakened other residents. A father who lives on the first floor opens his window and angrily asks the women/man to quieten down.

**S2**. A young woman/man is standing on a subway platform waiting for a train, which is running late. The platform is crowded with people. When the train finally arrives the woman/man makes a dash for the carriage. However, it is difficult to move in the crowd and people are jostling each other. This irritates the woman/man, who tries to push the people in front of her/him even harder, even though there is no room for them to move. The pushing makes the person in front of the woman/man lose balance and fall over. Other passengers look disapprovingly at her/him.

**S3**. A young woman/man is carrying a big sack of rubbish to the apartment block’s refuse-collection area. All the bins are completely full. Nevertheless, the woman/man still tries to force the sack into one of them. As a result, the sack breaks and litter spills out over the floor. The woman/man has an appointment, so she/he decides not to clean up the litter and simply to leave. As she/he is about to go she/he notices that the block’s janitor and some of its inhabitants have seen the whole incident. The janitor asks in a reproachful manner whether the woman/man intends to clean up the mess.

**S4**. A young woman/man is sitting in a library reading a book. Even though it is forbidden to bring food or drinks into the reading room, the woman/man has a cup of coffee with her/him. Deep in thought, she/he turns the pages of the book with one hand and carelessly handles the coffee cup with other, accidently spilling the coffee over the book. The book is soaked, the pages stick together and it is hard to make out what the text says. However, the woman/man has not yet borrowed the volume, so the damage cannot be associated with her/him. She/he decides quietly to put the book back on the shelf. Just when she/he is about to put it back she/he notices that the librarian is looking angrily at her/him from behind the desk. The librarian and a group of other people have seen the whole incident. The librarian asks if the woman/man intends to put the spoiled book back on the shelf.

**S5**. A young woman/man throws her/his jacket on a bench in a coffee shop to reserve the place. After purchasing coffee at the counter she/he notices that two people have sat down at that table. The pair’s lack of consideration makes the woman/man angry. She/he goes to the table and irritably states that she/he had reserved it. The two people look at the woman in amazement and give no indication that they will move. This reaction enrages the woman/man and she/he starts to remove the other people’s cups from the table. At this point the waiter steps in and asks the woman/man to come with him. He tells her/him that the two people had reserved the table before he/she came in, obviously not noticing their things on the table. The waiter then hands over the woman’s/man’s jacket and asks her/him to sit at a table at the back of the coffee shop.

**S6**. A young woman/man is walking to work. The work place is in a busy shopping street, which is full of tourists at this time of year. She/he soon needs to slow down to avoid bumping into a group of tourists who are walking in front of her/him. The woman/man tries again and again to get past the tourists. Eventually she/he becomes exasperated with their loitering and starts to push through them. As a result, the person at the front of the woman/man falls over. Passers-by look on in amazement at the woman's/man's behaviour, and several people and the tourist guide gaze angrily at her/him.

**Study 3**

**S1, less severe**. A young woman/man arrives at the front door of her/his apartment building late in the evening. The woman/man has a key to the door but it does not open right away. This makes the woman/man angry and she/he starts to pull the door handle with both hands and finally kicks it. The door opens but the pulling and the kick have broken the lock. In addition, the noise has awakened other residents. A father who lives on the first floor opens his window and angrily asks the women/man to quieten down.

**S1, more severe**. A young woman/man arrives at the front door of her/his apartment building late in the evening. The woman/man has a key to the door but it does not open right away. This makes the woman/man angry and she/he starts to violently kick it. After several kicks, the doors glass breaks and the door opens. In addition, the noise has awakened other residents. A father who lives on the first floor opens his window and angrily asks the women/man to quieten down.

**S2, less severe**. A young woman/man is standing on a subway platform waiting for a train, which is running late. The platform is crowded with people. When the train finally arrives the woman/man makes a dash for the carriage. However, it is difficult to move in the crowd and people are jostling each other. This irritates the woman/man, who tries to push the people in front of her/him even harder, even though there is no room for them to move. The pushing makes the person in front of the woman/man lose balance and fall over. Other passengers look disapprovingly at her/him.

**S2, more severe**. A young woman/man is standing on a subway platform waiting for a train, which is running late. The platform is crowded with people. When the train finally arrives the woman/man makes a dash for the carriage. However, it is difficult to move in the crowd and people are jostling each other. This irritates the woman/man, who tries to push the people in front of her/him even harder, even though there is no room for them to move. The pushing makes the person in front of the woman/man lose balance, fall over, and get severely injured. Other passengers look disapprovingly at her/him.

**S6, less severe**. A young woman/man is walking to work but is several minutes late for an important meeting. The work place is in a busy shopping street, which is full of tourists at this time of year. She/he soon needs to slow down to avoid bumping into a group of tourists who are walking in front of her/him. The woman/man tries again and again to get past the tourists. Eventually she/he becomes exasperated with their loitering and starts to push through them. As a result, the person at the front of the woman/man falls over. Passers-by look on in amazement at the woman's/man's behaviour, and several people and the tourist guide gaze angrily at her/him.

**S6, more severe**. A young woman/man is walking to work. The work place is in a busy shopping street, which is full of tourists at this time of year. She/he soon needs to slow down to avoid bumping into a group of tourists who are walking in front of her/him. The woman/man tries again and again to get past the tourists. Eventually she/he becomes exasperated with their loitering and starts to push through them. As a result, the person at the front of the woman/man falls over and get severely injured. Passers-by look on in amazement at the woman's/man's behaviour, and several people and the tourist guide gaze angrily at her/him.
