# Supplementary material for: How bodily expressions of emotion after norm violation influence perceivers’ moral judgments and prevent social exclusion: A socio-functional approach to nonverbal shame display
Source: PLoS One. 2020 Apr 30;15(4):e0232298. doi: 10.1371/journal.pone.0232298 (PMC7192454; doi:10.1371/journal.pone.0232298)
Supplement: S2 Appendix — (DOCX) [file pone.0232298.s002.docx]

**Study 1**

**Moral Sense** (1: Not at all, 9: Very much)

“The person understands that what s/he did was wrong”

**Conformity** (1: Not at all, 9: Very much)

“The person is willing to accept the consequences of his/her actions”

**Appropriateness** (1: Not at all, 9: Very much)

“The person’s reaction is appropriate in the situation”

**Valence, Arousal, and Dominance**

**
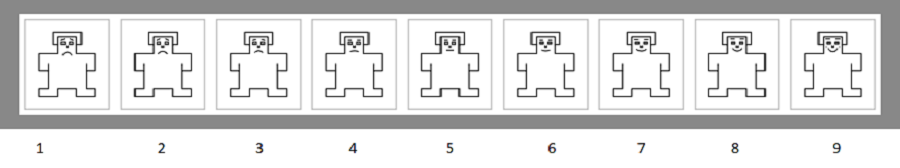
**


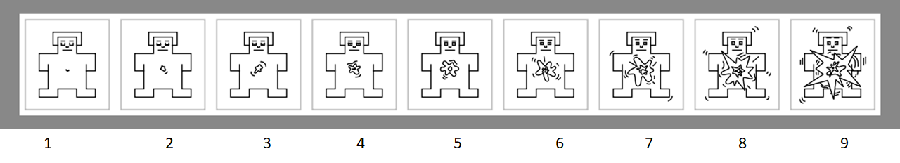


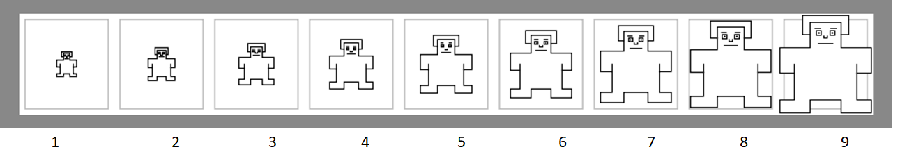


**Study 2**

**Empathy** (1: Not at all, 9: Very much)

“I would like to forgiveness towards the person”

“I would feel sympathy towards the person”

“I would feel concern towards the person”

**Moral Sense** (1: Not at all, 9: Very much)

“Has taken responsibility for what happened”

“Believes that s/he shouldn't have acted as s/he did”

“Believes that [breaking the rule] is wrong”

“REV: would probably behave in the same way in a similar situation if s/he was sure s/he would not get caught “

**Social Anxiety** (1: Not at all, 9: Very much)

“Is concerned that others will disapprove of her/him”

“Cares about others’ opinions of her/him”

“Believes that others might be angry with her/him”

**Intention to punish** (1: Not at all, 9: Very much)

“The person should be reprimanded”

“Disapproval of the person should be shown “

“Anger should be shown towards the person”

“The person should be punished in some way”

**Willingness to cooperate** (1: Not at all, 9: Very much)

“I could imagine myself cooperating with the person”

“REV:I would not like to be in touch with the person”

“REV:I would not choose the person as a work colleague”

“I could imagine myself choosing the person as a work colleague”

**Study 3**

Same as in Study 2.
