# Supplementary material for: How bodily expressions of emotion after norm violation influence perceivers’ moral judgments and prevent social exclusion: A socio-functional approach to nonverbal shame display
Source: PLoS One. 2020 Apr 30;15(4):e0232298. doi: 10.1371/journal.pone.0232298 (PMC7192454; doi:10.1371/journal.pone.0232298)
Supplement: S3 Appendix — (DOCX) [file pone.0232298.s003.docx]

Bivariate correlations between mediators and variance inflation factor and tolerance values (n=924)

|  | Moral Sense | Social Anxiety | Tolerance | VIF |
| --- | --- | --- | --- | --- |
| Moral Sense |  |  | 0.43 | 2.33 |
| Social Anxiety | .70* |  | 0.47 | 2.12 |
| Empathy | .47* | .40* | 0.77 | 1.30 |

*= p<.001. VIF refers to variance inflation factor

Levene’s test for homogeneity of variance based on mean (n=924)

| Model | Variable | Levene Statistic | df1 | df2 | p |
| --- | --- | --- | --- | --- | --- |
| Emotion + Severity + Emotion*Severity | Empathy | 1.55 | 7 | 916 | .15 |
|  | Punishment | 1.68 | 7 | 916 | .11 |
|  | Cooperation | 0.32 | 7 | 916 | .11 |
|  | Moral Sense | 0.31 | 7 | 916 | .94 |
|  | Social Anxiety | 1.94 | 7 | 916 | .06 |
| Emotion | Empathy | 0.33 | 3 | 920 | .81 |
|  | Punishment | 1.22 | 3 | 920 | .30 |
|  | Cooperation | 0.07 | 3 | 920 | .98 |
|  | Moral Sense | 0.19 | 3 | 920 | .90 |
|  | Social Anxiety | 1.67 | 3 | 920 | .17 |
| Severity | Empathy | 0.32 | 1 | 922 | .57 |
|  | Punishment | 3.21 | 1 | 922 | .07 |
|  | Cooperation | 1.64 | 1 | 922 | .20 |
|  | Moral Sense | 1.69 | 1 | 922 | .19 |
|  | Social Anxiety | 1.73 | 1 | 922 | .19 |
