# Supplementary material for: How bodily expressions of emotion after norm violation influence perceivers’ moral judgments and prevent social exclusion: A socio-functional approach to nonverbal shame display
Source: PLoS One. 2020 Apr 30;15(4):e0232298. doi: 10.1371/journal.pone.0232298 (PMC7192454; doi:10.1371/journal.pone.0232298)
Supplement: S4 Appendix — (DOCX) [file pone.0232298.s004.docx]

**Legend for variable names and models:**

**Variables**

moral_s = Moral Sense
social_a = Social Anxiety
empathy = Empathy
trans_s = Transgression Severity
cooper = Cooperation
punish = Punishment
X1 = Anger vs. Shame
X2 = Sadness vs. Shame
X3 = Neutral vs. Shame
Emotion = dummy coded emotion

**Models***

Model1 = Emotion –> Moral Sense -> Cooperation

Model2 = Emotion –> Social Anxiety -> Cooperation

Model3 = Emotion –> Empathy -> Cooperation

Model4 = Emotion –> Moral Sense -> Punishment

Model5 = Emotion –> Social Anxiety -> Punishment

Model6 = Emotion –> Empathy -> Punishment

Model7 = Emotion -> Empathy/Moral Sense/Social Anxiety -> Cooperation

Model8 = Emotion -> Empathy/Moral Sense/Social Anxiety -> Punishment

*Note that Transgression Severity is used as a covariate in all models

**INDIVIDUAL MEDIATION MODELS**

**Model1**

**M = Moral Sense // Y = Cooperation**

Run MATRIX procedure:


**************** PROCESS Procedure for SPSS Version 3.1 ******************


          Written by Andrew F. Hayes, Ph.D.       www.afhayes.com

    Documentation available in Hayes (2018). www.guilford.com/p/hayes3


**************************************************************************

Model  : 4

    Y  : cooper

    X  : Emotion

    M  : moral_s


Covariates:

 Trans_s


Sample

Size:  924


Coding of categorical X variable for analysis:

 Emotion       X1       X2       X3

    1,000     ,000     ,000     ,000

    2,000    1,000     ,000     ,000

    3,000     ,000    1,000     ,000

    4,000     ,000     ,000    1,000


**************************************************************************

OUTCOME VARIABLE:

 moral_s


Model Summary

          R       R-sq        MSE          F        df1        df2          p

      ,4878      ,2379     2,5887    71,7365     4,0000   919,0000      ,0000


Model

              coeff         se          t          p       LLCI       ULCI

constant     6,0989      ,1898    32,1408      ,0000     5,7265     6,4714

X1          -1,8765      ,1470   -12,7661      ,0000    -2,1649    -1,5880

X2           -,3657      ,1512    -2,4185      ,0158     -,6624     -,0689

X3          -2,0051      ,1482   -13,5261      ,0000    -2,2961    -1,7142

Trans_s        -,1816      ,1072    -1,6939      ,0906     -,3919      ,0288


Covariance matrix of regression parameter estimates:

           constant         X1         X2         X3      Trans_s

constant      ,0360     -,0115     -,0123     -,0120     -,0171

X1           -,0115      ,0216      ,0107      ,0107      ,0006

X2           -,0123      ,0107      ,0229      ,0107      ,0011

X3           -,0120      ,0107      ,0107      ,0220      ,0009

Trans_s        -,0171      ,0006      ,0011      ,0009      ,0115


**************************************************************************

OUTCOME VARIABLE:

 cooper


Model Summary

          R       R-sq        MSE          F        df1        df2          p

      ,4498      ,2023     3,1055    46,5591     5,0000   918,0000      ,0000


Model

              coeff         se          t          p       LLCI       ULCI

constant     3,2768      ,3029    10,8176      ,0000     2,6823     3,8712

X1           -,0110      ,1747     -,0632      ,9496     -,3539      ,3318

X2           -,1188      ,1661     -,7149      ,4749     -,4448      ,2073

X3           -,0484      ,1778     -,2724      ,7854     -,3974      ,3005

moral_s      ,4544      ,0361    12,5775      ,0000      ,3835      ,5253

Trans_s        -,5483      ,1176    -4,6635      ,0000     -,7791     -,3176


Covariance matrix of regression parameter estimates:

           constant         X1         X2         X3   moral_s      Trans_s

constant      ,0918     -,0288     -,0177     -,0303     -,0080     -,0219

X1           -,0288      ,0305      ,0137      ,0177      ,0024      ,0011

X2           -,0177      ,0137      ,0276      ,0138      ,0005      ,0014

X3           -,0303      ,0177      ,0138      ,0316      ,0026      ,0015

moral_s     -,0080      ,0024      ,0005      ,0026      ,0013      ,0002

Trans_s        -,0219      ,0011      ,0014      ,0015      ,0002      ,0138


************************** TOTAL EFFECT MODEL ****************************

OUTCOME VARIABLE:

 cooper


Model Summary

          R       R-sq        MSE          F        df1        df2          p

      ,2546      ,0648     3,6367    15,9264     4,0000   919,0000      ,0000


Model

              coeff         se          t          p       LLCI       ULCI

constant     6,0483      ,2249    26,8917      ,0000     5,6069     6,4897

X1           -,8637      ,1742    -4,9578      ,0000    -1,2057     -,5218

X2           -,2849      ,1792    -1,5899      ,1122     -,6366      ,0668

X3           -,9596      ,1757    -5,4615      ,0000    -1,3045     -,6148

Trans_s        -,6308      ,1270    -4,9656      ,0000     -,8802     -,3815


Covariance matrix of regression parameter estimates:

           constant         X1         X2         X3      Trans_s

constant      ,0506     -,0162     -,0173     -,0168     -,0240

X1           -,0162      ,0304      ,0150      ,0150      ,0008

X2           -,0173      ,0150      ,0321      ,0151      ,0016

X3           -,0168      ,0150      ,0151      ,0309      ,0012

Trans_s        -,0240      ,0008      ,0016      ,0012      ,0161


************** TOTAL, DIRECT, AND INDIRECT EFFECTS OF X ON Y **************


Relative total effects of X on Y:

       Effect         se          t          p       LLCI       ULCI       c_ps

X1     -,8637      ,1742    -4,9578      ,0000    -1,2057     -,5218     -,4390

X2     -,2849      ,1792    -1,5899      ,1122     -,6366      ,0668     -,1448

X3     -,9596      ,1757    -5,4615      ,0000    -1,3045     -,6148     -,4877


Omnibus test of total effect of X on Y:

    R2-chng          F        df1        df2          p

      ,0419    13,7263     3,0000   919,0000      ,0000

----------


Relative direct effects of X on Y

       Effect         se          t          p       LLCI       ULCI      c'_ps

X1     -,0110      ,1747     -,0632      ,9496     -,3539      ,3318     -,0056

X2     -,1188      ,1661     -,7149      ,4749     -,4448      ,2073     -,0604

X3     -,0484      ,1778     -,2724      ,7854     -,3974      ,3005     -,0246


Omnibus test of direct effect of X on Y:

    R2-chng          F        df1        df2          p

      ,0005      ,2050     3,0000   918,0000      ,8930

----------


Relative indirect effects of X on Y


 Emotion    ->    moral_s    ->    cooper


       Effect     BootSE   BootLLCI   BootULCI

X1     -,8527      ,1028    -1,0687     -,6674

X2     -,1662      ,0710     -,3115     -,0325

X3     -,9112      ,1090    -1,1370     -,7126


Partially standardized relative indirect effect(s) of X on Y:


 Emotion    ->    moral_s    ->    cooper


       Effect     BootSE   BootLLCI   BootULCI

X1     -,4333      ,0503     -,5369     -,3423

X2     -,0844      ,0359     -,1573     -,0168

X3     -,4631      ,0532     -,5733     -,3635


*********** BOOTSTRAP RESULTS FOR REGRESSION MODEL PARAMETERS ************


OUTCOME VARIABLE:

 moral_s


              Coeff   BootMean     BootSE   BootLLCI   BootULCI

constant     6,0989     6,0991      ,1856     5,7402     6,4645

X1          -1,8765    -1,8762      ,1435    -2,1636    -1,6027

X2           -,3657     -,3672      ,1510     -,6670     -,0746

X3          -2,0051    -2,0060      ,1463    -2,2936    -1,7192

Trans_s        -,1816     -,1814      ,1079     -,3934      ,0266


----------


OUTCOME VARIABLE:

 cooper


              Coeff   BootMean     BootSE   BootLLCI   BootULCI

constant     3,2768     3,2700      ,3238     2,6365     3,9014

X1           -,0110     -,0083      ,1816     -,3742      ,3489

X2           -,1188     -,1171      ,1658     -,4465      ,2081

X3           -,0484     -,0475      ,1860     -,4156      ,3099

moral_s      ,4544      ,4552      ,0387      ,3777      ,5298

Trans_s        -,5483     -,5475      ,1178     -,7821     -,3164


*********************** ANALYSIS NOTES AND ERRORS ************************


Level of confidence for all confidence intervals in output:

  95,0000


Number of bootstrap samples for percentile bootstrap confidence intervals:

  5000


NOTE: Variables names longer than eight characters can produce incorrect output.

      Shorter variable names are recommended.


------ END MATRIX -----

**Model2**

**M = Social Anxiety // Y = Cooperation**

Run MATRIX procedure:


**************** PROCESS Procedure for SPSS Version 3.1 ******************


          Written by Andrew F. Hayes, Ph.D.       www.afhayes.com

    Documentation available in Hayes (2018). www.guilford.com/p/hayes3


**************************************************************************

Model  : 4

    Y  : cooper

    X  : Emotion

    M  : social_a


Covariates:

 Trans_s


Sample

Size:  924


Coding of categorical X variable for analysis:

 Emotion       X1       X2       X3

    1,000     ,000     ,000     ,000

    2,000    1,000     ,000     ,000

    3,000     ,000    1,000     ,000

    4,000     ,000     ,000    1,000


**************************************************************************

OUTCOME VARIABLE:

 social_a


Model Summary

          R       R-sq        MSE          F        df1        df2          p

      ,4987      ,2487     2,9707    76,0588     4,0000   919,0000      ,0000


Model

              coeff         se          t          p       LLCI       ULCI

constant     6,7903      ,2033    33,4038      ,0000     6,3914     7,1892

X1          -1,5802      ,1575   -10,0354      ,0000    -1,8892    -1,2712

X2           -,1232      ,1620     -,7606      ,4471     -,4411      ,1947

X3          -2,3386      ,1588   -14,7262      ,0000    -2,6503    -2,0269

Trans_s        -,2023      ,1148    -1,7621      ,0784     -,4277      ,0230


Covariance matrix of regression parameter estimates:

           constant         X1         X2         X3      Trans_s

constant      ,0413     -,0132     -,0141     -,0137     -,0196

X1           -,0132      ,0248      ,0123      ,0123      ,0007

X2           -,0141      ,0123      ,0262      ,0123      ,0013

X3           -,0137      ,0123      ,0123      ,0252      ,0010

Trans_s        -,0196      ,0007      ,0013      ,0010      ,0132


**************************************************************************

OUTCOME VARIABLE:

 cooper


Model Summary

          R       R-sq        MSE          F        df1        df2          p

      ,3596      ,1293     3,3895    27,2735     5,0000   918,0000      ,0000


Model

              coeff         se          t          p       LLCI       ULCI

constant     4,0751      ,3231    12,6125      ,0000     3,4410     4,7092

X1           -,4046      ,1772    -2,2834      ,0226     -,7523     -,0568

X2           -,2491      ,1731    -1,4395      ,1503     -,5888      ,0905

X3           -,2800      ,1886    -1,4849      ,1379     -,6501      ,0901

social_a      ,2906      ,0352     8,2472      ,0000      ,2214      ,3597

Trans_s        -,5720      ,1229    -4,6562      ,0000     -,8132     -,3309


Covariance matrix of regression parameter estimates:

           constant         X1         X2         X3   social_a      Trans_s

constant      ,1044     -,0284     -,0172     -,0354     -,0084     -,0241

X1           -,0284      ,0314      ,0143      ,0186      ,0020      ,0012

X2           -,0172      ,0143      ,0299      ,0144      ,0002      ,0015

X3           -,0354      ,0186      ,0144      ,0356      ,0029      ,0017

social_a     -,0084      ,0020      ,0002      ,0029      ,0012      ,0003

Trans_s        -,0241      ,0012      ,0015      ,0017      ,0003      ,0151


************************** TOTAL EFFECT MODEL ****************************

OUTCOME VARIABLE:

 cooper


Model Summary

          R       R-sq        MSE          F        df1        df2          p

      ,2546      ,0648     3,6367    15,9264     4,0000   919,0000      ,0000


Model

              coeff         se          t          p       LLCI       ULCI

constant     6,0483      ,2249    26,8917      ,0000     5,6069     6,4897

X1           -,8637      ,1742    -4,9578      ,0000    -1,2057     -,5218

X2           -,2849      ,1792    -1,5899      ,1122     -,6366      ,0668

X3           -,9596      ,1757    -5,4615      ,0000    -1,3045     -,6148

Trans_s        -,6308      ,1270    -4,9656      ,0000     -,8802     -,3815


Covariance matrix of regression parameter estimates:

           constant         X1         X2         X3      Trans_s

constant      ,0506     -,0162     -,0173     -,0168     -,0240

X1           -,0162      ,0304      ,0150      ,0150      ,0008

X2           -,0173      ,0150      ,0321      ,0151      ,0016

X3           -,0168      ,0150      ,0151      ,0309      ,0012

Trans_s        -,0240      ,0008      ,0016      ,0012      ,0161


************** TOTAL, DIRECT, AND INDIRECT EFFECTS OF X ON Y **************


Relative total effects of X on Y:

       Effect         se          t          p       LLCI       ULCI       c_ps

X1     -,8637      ,1742    -4,9578      ,0000    -1,2057     -,5218     -,4390

X2     -,2849      ,1792    -1,5899      ,1122     -,6366      ,0668     -,1448

X3     -,9596      ,1757    -5,4615      ,0000    -1,3045     -,6148     -,4877


Omnibus test of total effect of X on Y:

    R2-chng          F        df1        df2          p

      ,0419    13,7263     3,0000   919,0000      ,0000

----------


Relative direct effects of X on Y

       Effect         se          t          p       LLCI       ULCI      c'_ps

X1     -,4046      ,1772    -2,2834      ,0226     -,7523     -,0568     -,2056

X2     -,2491      ,1731    -1,4395      ,1503     -,5888      ,0905     -,1266

X3     -,2800      ,1886    -1,4849      ,1379     -,6501      ,0901     -,1423


Omnibus test of direct effect of X on Y:

    R2-chng          F        df1        df2          p

      ,0051     1,8067     3,0000   918,0000      ,1443

----------


Relative indirect effects of X on Y


 Emotion    ->    social_a    ->    cooper


       Effect     BootSE   BootLLCI   BootULCI

X1     -,4592      ,0785     -,6227     -,3147

X2     -,0358      ,0455     -,1251      ,0510

X3     -,6796      ,1010     -,8890     -,4910


Partially standardized relative indirect effect(s) of X on Y:


 Emotion    ->    social_a    ->    cooper


       Effect     BootSE   BootLLCI   BootULCI

X1     -,2334      ,0391     -,3148     -,1613

X2     -,0182      ,0231     -,0636      ,0260

X3     -,3454      ,0501     -,4488     -,2513


*********** BOOTSTRAP RESULTS FOR REGRESSION MODEL PARAMETERS ************


OUTCOME VARIABLE:

 social_a


              Coeff   BootMean     BootSE   BootLLCI   BootULCI

constant     6,7903     6,7920      ,2031     6,3942     7,1870

X1          -1,5802    -1,5794      ,1640    -1,8976    -1,2597

X2           -,1232     -,1243      ,1541     -,4137      ,1730

X3          -2,3386    -2,3401      ,1575    -2,6422    -2,0268

Trans_s        -,2023     -,2026      ,1157     -,4294      ,0230


----------


OUTCOME VARIABLE:

 cooper


              Coeff   BootMean     BootSE   BootLLCI   BootULCI

constant     4,0751     4,0706      ,3297     3,4187     4,7159

X1           -,4046     -,3989      ,1781     -,7494     -,0472

X2           -,2491     -,2474      ,1740     -,5962      ,0960

X3           -,2800     -,2776      ,1918     -,6779      ,0872

social_a      ,2906      ,2911      ,0374      ,2181      ,3652

Trans_s        -,5720     -,5721      ,1243     -,8189     -,3305


*********************** ANALYSIS NOTES AND ERRORS ************************


Level of confidence for all confidence intervals in output:

  95,0000


Number of bootstrap samples for percentile bootstrap confidence intervals:

  5000


NOTE: Variables names longer than eight characters can produce incorrect output.

      Shorter variable names are recommended.


------ END MATRIX -----

**Model3**

**M = Empathy // Y = Cooperation**

Run MATRIX procedure:


**************** PROCESS Procedure for SPSS Version 3.1 ******************


          Written by Andrew F. Hayes, Ph.D.       www.afhayes.com

    Documentation available in Hayes (2018). www.guilford.com/p/hayes3


**************************************************************************

Model  : 4

    Y  : cooper

    X  : Emotion

    M  : empathy


Covariates:

 Trans_s


Sample

Size:  924


Coding of categorical X variable for analysis:

 Emotion       X1       X2       X3

    1,000     ,000     ,000     ,000

    2,000    1,000     ,000     ,000

    3,000     ,000    1,000     ,000

    4,000     ,000     ,000    1,000


**************************************************************************

OUTCOME VARIABLE:

 empathy


Model Summary

          R       R-sq        MSE          F        df1        df2          p

      ,2982      ,0889     3,6172    22,4180     4,0000   919,0000      ,0000


Model

              coeff         se          t          p       LLCI       ULCI

constant     6,0848      ,2243    27,1266      ,0000     5,6446     6,5250

X1           -,7681      ,1738    -4,4204      ,0000    -1,1091     -,4271

X2           -,1076      ,1787     -,6019      ,5474     -,4583      ,2432

X3           -,9825      ,1752    -5,6066      ,0000    -1,3264     -,6386

Trans_s        -,8669      ,1267    -6,8417      ,0000    -1,1155     -,6182


Covariance matrix of regression parameter estimates:

           constant         X1         X2         X3      Trans_s

constant      ,0503     -,0161     -,0172     -,0167     -,0238

X1           -,0161      ,0302      ,0150      ,0149      ,0008

X2           -,0172      ,0150      ,0319      ,0150      ,0016

X3           -,0167      ,0149      ,0150      ,0307      ,0012

Trans_s        -,0238      ,0008      ,0016      ,0012      ,0161


**************************************************************************

OUTCOME VARIABLE:

 cooper


Model Summary

          R       R-sq        MSE          F        df1        df2          p

      ,5819      ,3386     2,5750    93,9728     5,0000   918,0000      ,0000


Model

              coeff         se          t          p       LLCI       ULCI

constant     2,7475      ,2540    10,8183      ,0000     2,2490     3,2459

X1           -,4471      ,1482    -3,0179      ,0026     -,7379     -,1563

X2           -,2266      ,1508    -1,5022      ,1334     -,5226      ,0694

X3           -,4267      ,1504    -2,8376      ,0046     -,7217     -,1316

empathy       ,5425      ,0278    19,4908      ,0000      ,4878      ,5971

Trans_s        -,1606      ,1096    -1,4654      ,1432     -,3757      ,0545


Covariance matrix of regression parameter estimates:

           constant         X1         X2         X3    empathy      Trans_s

constant      ,0645     -,0151     -,0128     -,0165     -,0047     -,0211

X1           -,0151      ,0219      ,0107      ,0112      ,0006      ,0011

X2           -,0128      ,0107      ,0227      ,0108      ,0001      ,0012

X3           -,0165      ,0112      ,0108      ,0226      ,0008      ,0015

empathy      -,0047      ,0006      ,0001      ,0008      ,0008      ,0007

Trans_s        -,0211      ,0011      ,0012      ,0015      ,0007      ,0120


************************** TOTAL EFFECT MODEL ****************************

OUTCOME VARIABLE:

 cooper


Model Summary

          R       R-sq        MSE          F        df1        df2          p

      ,2546      ,0648     3,6367    15,9264     4,0000   919,0000      ,0000


Model

              coeff         se          t          p       LLCI       ULCI

constant     6,0483      ,2249    26,8917      ,0000     5,6069     6,4897

X1           -,8637      ,1742    -4,9578      ,0000    -1,2057     -,5218

X2           -,2849      ,1792    -1,5899      ,1122     -,6366      ,0668

X3           -,9596      ,1757    -5,4615      ,0000    -1,3045     -,6148

Trans_s        -,6308      ,1270    -4,9656      ,0000     -,8802     -,3815


Covariance matrix of regression parameter estimates:

           constant         X1         X2         X3      Trans_s

constant      ,0506     -,0162     -,0173     -,0168     -,0240

X1           -,0162      ,0304      ,0150      ,0150      ,0008

X2           -,0173      ,0150      ,0321      ,0151      ,0016

X3           -,0168      ,0150      ,0151      ,0309      ,0012

Trans_s        -,0240      ,0008      ,0016      ,0012      ,0161


************** TOTAL, DIRECT, AND INDIRECT EFFECTS OF X ON Y **************


Relative total effects of X on Y:

       Effect         se          t          p       LLCI       ULCI       c_ps

X1     -,8637      ,1742    -4,9578      ,0000    -1,2057     -,5218     -,4390

X2     -,2849      ,1792    -1,5899      ,1122     -,6366      ,0668     -,1448

X3     -,9596      ,1757    -5,4615      ,0000    -1,3045     -,6148     -,4877


Omnibus test of total effect of X on Y:

    R2-chng          F        df1        df2          p

      ,0419    13,7263     3,0000   919,0000      ,0000

----------


Relative direct effects of X on Y

       Effect         se          t          p       LLCI       ULCI      c'_ps

X1     -,4471      ,1482    -3,0179      ,0026     -,7379     -,1563     -,2272

X2     -,2266      ,1508    -1,5022      ,1334     -,5226      ,0694     -,1151

X3     -,4267      ,1504    -2,8376      ,0046     -,7217     -,1316     -,2168


Omnibus test of direct effect of X on Y:

    R2-chng          F        df1        df2          p

      ,0084     3,8712     3,0000   918,0000      ,0091

----------


Relative indirect effects of X on Y


 Emotion    ->    empathy     ->    cooper


       Effect     BootSE   BootLLCI   BootULCI

X1     -,4166      ,0971     -,6082     -,2280

X2     -,0584      ,0997     -,2579      ,1392

X3     -,5330      ,1015     -,7400     -,3403


Partially standardized relative indirect effect(s) of X on Y:


 Emotion    ->    empathy     ->    cooper


       Effect     BootSE   BootLLCI   BootULCI

X1     -,2117      ,0486     -,3058     -,1163

X2     -,0297      ,0506     -,1304      ,0713

X3     -,2709      ,0505     -,3731     -,1738


*********** BOOTSTRAP RESULTS FOR REGRESSION MODEL PARAMETERS ************


OUTCOME VARIABLE:

 empathy


              Coeff   BootMean     BootSE   BootLLCI   BootULCI

constant     6,0848     6,0852      ,2241     5,6369     6,5299

X1           -,7681     -,7655      ,1739    -1,1051     -,4248

X2           -,1076     -,1083      ,1835     -,4763      ,2578

X3           -,9825     -,9824      ,1769    -1,3377     -,6329

Trans_s        -,8669     -,8665      ,1271    -1,1149     -,6139


----------


OUTCOME VARIABLE:

 cooper


              Coeff   BootMean     BootSE   BootLLCI   BootULCI

constant     2,7475     2,7523      ,2676     2,2354     3,2845

X1           -,4471     -,4473      ,1482     -,7461     -,1601

X2           -,2266     -,2242      ,1531     -,5279      ,0864

X3           -,4267     -,4250      ,1497     -,7184     -,1258

empathy       ,5425      ,5424      ,0298      ,4847      ,6013

Trans_s        -,1606     -,1643      ,1107     -,3804      ,0535


*********************** ANALYSIS NOTES AND ERRORS ************************


Level of confidence for all confidence intervals in output:

  95,0000


Number of bootstrap samples for percentile bootstrap confidence intervals:

  5000


------ END MATRIX -----

**Model4**

**M = Moral Sense // Y = Punishment**

Run MATRIX procedure:


**************** PROCESS Procedure for SPSS Version 3.1 ******************


          Written by Andrew F. Hayes, Ph.D.       www.afhayes.com

    Documentation available in Hayes (2018). www.guilford.com/p/hayes3


**************************************************************************

Model  : 4

    Y  : punish

    X  : Emotion

    M  : moral_s


Covariates:

 Trans_s


Sample

Size:  924


Coding of categorical X variable for analysis:

 Emotion       X1       X2       X3

    1,000     ,000     ,000     ,000

    2,000    1,000     ,000     ,000

    3,000     ,000    1,000     ,000

    4,000     ,000     ,000    1,000


**************************************************************************

OUTCOME VARIABLE:

 moral_s


Model Summary

          R       R-sq        MSE          F        df1        df2          p

      ,4878      ,2379     2,5887    71,7365     4,0000   919,0000      ,0000


Model

              coeff         se          t          p       LLCI       ULCI

constant     6,0989      ,1898    32,1408      ,0000     5,7265     6,4714

X1          -1,8765      ,1470   -12,7661      ,0000    -2,1649    -1,5880

X2           -,3657      ,1512    -2,4185      ,0158     -,6624     -,0689

X3          -2,0051      ,1482   -13,5261      ,0000    -2,2961    -1,7142

Trans_s        -,1816      ,1072    -1,6939      ,0906     -,3919      ,0288


Covariance matrix of regression parameter estimates:

           constant         X1         X2         X3      Trans_s

constant      ,0360     -,0115     -,0123     -,0120     -,0171

X1           -,0115      ,0216      ,0107      ,0107      ,0006

X2           -,0123      ,0107      ,0229      ,0107      ,0011

X3           -,0120      ,0107      ,0107      ,0220      ,0009

Trans_s        -,0171      ,0006      ,0011      ,0009      ,0115


**************************************************************************

OUTCOME VARIABLE:

 punish


Model Summary

          R       R-sq        MSE          F        df1        df2          p

      ,3772      ,1423     2,7149    30,4619     5,0000   918,0000      ,0000


Model

              coeff         se          t          p       LLCI       ULCI

constant     4,8363      ,2832    17,0760      ,0000     4,2805     5,3921

X1           -,1633      ,1633     -,9997      ,3177     -,4838      ,1573

X2            ,0297      ,1553      ,1912      ,8484     -,2751      ,3345

X3           -,2470      ,1662    -1,4858      ,1377     -,5733      ,0793

moral_s     -,2723      ,0338    -8,0616      ,0000     -,3386     -,2060

Trans_s         ,9440      ,1099     8,5869      ,0000      ,7283     1,1598


Covariance matrix of regression parameter estimates:

           constant         X1         X2         X3   moral_s      Trans_s

constant      ,0802     -,0251     -,0155     -,0265     -,0070     -,0192

X1           -,0251      ,0267      ,0120      ,0155      ,0021      ,0010

X2           -,0155      ,0120      ,0241      ,0121      ,0004      ,0013

X3           -,0265      ,0155      ,0121      ,0276      ,0023      ,0013

moral_s     -,0070      ,0021      ,0004      ,0023      ,0011      ,0002

Trans_s        -,0192      ,0010      ,0013      ,0013      ,0002      ,0121


************************** TOTAL EFFECT MODEL ****************************

OUTCOME VARIABLE:

 punish


Model Summary

          R       R-sq        MSE          F        df1        df2          p

      ,2856      ,0816     2,9040    20,4089     4,0000   919,0000      ,0000


Model

              coeff         se          t          p       LLCI       ULCI

constant     3,1753      ,2010    15,7991      ,0000     2,7809     3,5698

X1            ,3477      ,1557     2,2337      ,0257      ,0422      ,6533

X2            ,1293      ,1601      ,8073      ,4197     -,1850      ,4436

X3            ,2991      ,1570     1,9048      ,0571     -,0091      ,6072

Trans_s         ,9935      ,1135     8,7512      ,0000      ,7707     1,2163


Covariance matrix of regression parameter estimates:

           constant         X1         X2         X3      Trans_s

constant      ,0404     -,0129     -,0138     -,0134     -,0191

X1           -,0129      ,0242      ,0120      ,0120      ,0007

X2           -,0138      ,0120      ,0256      ,0120      ,0013

X3           -,0134      ,0120      ,0120      ,0247      ,0010

Trans_s        -,0191      ,0007      ,0013      ,0010      ,0129


************** TOTAL, DIRECT, AND INDIRECT EFFECTS OF X ON Y **************


Relative total effects of X on Y:

       Effect         se          t          p       LLCI       ULCI       c_ps

X1      ,3477      ,1557     2,2337      ,0257      ,0422      ,6533      ,1960

X2      ,1293      ,1601      ,8073      ,4197     -,1850      ,4436      ,0729

X3      ,2991      ,1570     1,9048      ,0571     -,0091      ,6072      ,1686


Omnibus test of total effect of X on Y:

    R2-chng          F        df1        df2          p

      ,0062     2,0843     3,0000   919,0000      ,1007

----------


Relative direct effects of X on Y

       Effect         se          t          p       LLCI       ULCI      c'_ps

X1     -,1633      ,1633     -,9997      ,3177     -,4838      ,1573     -,0920

X2      ,0297      ,1553      ,1912      ,8484     -,2751      ,3345      ,0167

X3     -,2470      ,1662    -1,4858      ,1377     -,5733      ,0793     -,1392


Omnibus test of direct effect of X on Y:

    R2-chng          F        df1        df2          p

      ,0032     1,1520     3,0000   918,0000      ,3271

----------


Relative indirect effects of X on Y


 Emotion    ->    moral_s    ->    punish


       Effect     BootSE   BootLLCI   BootULCI

X1      ,5110      ,0796      ,3643      ,6724

X2      ,0996      ,0449      ,0166      ,1947

X3      ,5461      ,0835      ,3881      ,7160


Partially standardized relative indirect effect(s) of X on Y:


 Emotion    ->    moral_s    ->    punish


       Effect     BootSE   BootLLCI   BootULCI

X1      ,2880      ,0442      ,2058      ,3785

X2      ,0561      ,0253      ,0096      ,1092

X3      ,3078      ,0463      ,2201      ,4015


*********** BOOTSTRAP RESULTS FOR REGRESSION MODEL PARAMETERS ************


OUTCOME VARIABLE:

 moral_s


              Coeff   BootMean     BootSE   BootLLCI   BootULCI

constant     6,0989     6,1013      ,1866     5,7354     6,4682

X1          -1,8765    -1,8765      ,1462    -2,1541    -1,5849

X2           -,3657     -,3636      ,1528     -,6635     -,0633

X3          -2,0051    -2,0092      ,1493    -2,2977    -1,7085

Trans_s        -,1816     -,1828      ,1078     -,3907      ,0250


----------


OUTCOME VARIABLE:

 punish


              Coeff   BootMean     BootSE   BootLLCI   BootULCI

constant     4,8363     4,8320      ,2960     4,2382     5,4158

X1           -,1633     -,1596      ,1754     -,5017      ,1860

X2            ,0297      ,0305      ,1562     -,2706      ,3371

X3           -,2470     -,2433      ,1692     -,5746      ,0930

moral_s     -,2723     -,2719      ,0354     -,3415     -,2031

Trans_s         ,9440      ,9450      ,1134      ,7191     1,1656


*********************** ANALYSIS NOTES AND ERRORS ************************


Level of confidence for all confidence intervals in output:

  95,0000


Number of bootstrap samples for percentile bootstrap confidence intervals:

  5000


NOTE: Variables names longer than eight characters can produce incorrect output.

      Shorter variable names are recommended.


------ END MATRIX -----

**Model5**

**M = Social Anxiety // Y = Punishment**

Run MATRIX procedure:


**************** PROCESS Procedure for SPSS Version 3.1 ******************


          Written by Andrew F. Hayes, Ph.D.       www.afhayes.com

    Documentation available in Hayes (2018). www.guilford.com/p/hayes3


**************************************************************************

Model  : 4

    Y  : punish

    X  : Emotion

    M  : social_a


Covariates:

 Trans_s


Sample

Size:  924


Coding of categorical X variable for analysis:

 Emotion       X1       X2       X3

    1,000     ,000     ,000     ,000

    2,000    1,000     ,000     ,000

    3,000     ,000    1,000     ,000

    4,000     ,000     ,000    1,000


**************************************************************************

OUTCOME VARIABLE:

 social_a


Model Summary

          R       R-sq        MSE          F        df1        df2          p

      ,4987      ,2487     2,9707    76,0588     4,0000   919,0000      ,0000


Model

              coeff         se          t          p       LLCI       ULCI

constant     6,7903      ,2033    33,4038      ,0000     6,3914     7,1892

X1          -1,5802      ,1575   -10,0354      ,0000    -1,8892    -1,2712

X2           -,1232      ,1620     -,7606      ,4471     -,4411      ,1947

X3          -2,3386      ,1588   -14,7262      ,0000    -2,6503    -2,0269

Trans_s        -,2023      ,1148    -1,7621      ,0784     -,4277      ,0230


Covariance matrix of regression parameter estimates:

           constant         X1         X2         X3      Trans_s

constant      ,0413     -,0132     -,0141     -,0137     -,0196

X1           -,0132      ,0248      ,0123      ,0123      ,0007

X2           -,0141      ,0123      ,0262      ,0123      ,0013

X3           -,0137      ,0123      ,0123      ,0252      ,0010

Trans_s        -,0196      ,0007      ,0013      ,0010      ,0132


**************************************************************************

OUTCOME VARIABLE:

 punish


Model Summary

          R       R-sq        MSE          F        df1        df2          p

      ,3076      ,0946     2,8659    19,1880     5,0000   918,0000      ,0000


Model

              coeff         se          t          p       LLCI       ULCI

constant     3,9752      ,2971    13,3803      ,0000     3,3921     4,5583

X1            ,1616      ,1629      ,9919      ,3215     -,1581      ,4813

X2            ,1148      ,1591      ,7212      ,4709     -,1975      ,4271

X3            ,0236      ,1734      ,1361      ,8918     -,3167      ,3639

social_a     -,1178      ,0324    -3,6358      ,0003     -,1814     -,0542

Trans_s         ,9696      ,1130     8,5834      ,0000      ,7479     1,1913


Covariance matrix of regression parameter estimates:

           constant         X1         X2         X3   social_a      Trans_s

constant      ,0883     -,0240     -,0145     -,0299     -,0071     -,0203

X1           -,0240      ,0265      ,0121      ,0157      ,0017      ,0010

X2           -,0145      ,0121      ,0253      ,0122      ,0001      ,0013

X3           -,0299      ,0157      ,0122      ,0301      ,0025      ,0015

social_a     -,0071      ,0017      ,0001      ,0025      ,0010      ,0002

Trans_s        -,0203      ,0010      ,0013      ,0015      ,0002      ,0128


************************** TOTAL EFFECT MODEL ****************************

OUTCOME VARIABLE:

 punish


Model Summary

          R       R-sq        MSE          F        df1        df2          p

      ,2856      ,0816     2,9040    20,4089     4,0000   919,0000      ,0000


Model

              coeff         se          t          p       LLCI       ULCI

constant     3,1753      ,2010    15,7991      ,0000     2,7809     3,5698

X1            ,3477      ,1557     2,2337      ,0257      ,0422      ,6533

X2            ,1293      ,1601      ,8073      ,4197     -,1850      ,4436

X3            ,2991      ,1570     1,9048      ,0571     -,0091      ,6072

Trans_s         ,9935      ,1135     8,7512      ,0000      ,7707     1,2163


Covariance matrix of regression parameter estimates:

           constant         X1         X2         X3      Trans_s

constant      ,0404     -,0129     -,0138     -,0134     -,0191

X1           -,0129      ,0242      ,0120      ,0120      ,0007

X2           -,0138      ,0120      ,0256      ,0120      ,0013

X3           -,0134      ,0120      ,0120      ,0247      ,0010

Trans_s        -,0191      ,0007      ,0013      ,0010      ,0129


************** TOTAL, DIRECT, AND INDIRECT EFFECTS OF X ON Y **************


Relative total effects of X on Y:

       Effect         se          t          p       LLCI       ULCI       c_ps

X1      ,3477      ,1557     2,2337      ,0257      ,0422      ,6533      ,1960

X2      ,1293      ,1601      ,8073      ,4197     -,1850      ,4436      ,0729

X3      ,2991      ,1570     1,9048      ,0571     -,0091      ,6072      ,1686


Omnibus test of total effect of X on Y:

    R2-chng          F        df1        df2          p

      ,0062     2,0843     3,0000   919,0000      ,1007

----------


Relative direct effects of X on Y

       Effect         se          t          p       LLCI       ULCI      c'_ps

X1      ,1616      ,1629      ,9919      ,3215     -,1581      ,4813      ,0911

X2      ,1148      ,1591      ,7212      ,4709     -,1975      ,4271      ,0647

X3      ,0236      ,1734      ,1361      ,8918     -,3167      ,3639      ,0133


Omnibus test of direct effect of X on Y:

    R2-chng          F        df1        df2          p

      ,0014      ,4736     3,0000   918,0000      ,7007

----------


Relative indirect effects of X on Y


 Emotion    ->    social_a    ->    punish


       Effect     BootSE   BootLLCI   BootULCI

X1      ,1861      ,0581      ,0794      ,3061

X2      ,0145      ,0201     -,0225      ,0583

X3      ,2755      ,0814      ,1193      ,4433


Partially standardized relative indirect effect(s) of X on Y:


 Emotion    ->    social_a    ->    punish


       Effect     BootSE   BootLLCI   BootULCI

X1      ,1049      ,0327      ,0449      ,1738

X2      ,0082      ,0113     -,0127      ,0330

X3      ,1553      ,0458      ,0674      ,2494


*********** BOOTSTRAP RESULTS FOR REGRESSION MODEL PARAMETERS ************


OUTCOME VARIABLE:

 social_a


              Coeff   BootMean     BootSE   BootLLCI   BootULCI

constant     6,7903     6,7922      ,2008     6,3903     7,1813

X1          -1,5802    -1,5839      ,1609    -1,8993    -1,2654

X2           -,1232     -,1230      ,1560     -,4276      ,1844

X3          -2,3386    -2,3408      ,1606    -2,6552    -2,0218

Trans_s        -,2023     -,2025      ,1152     -,4281      ,0220


----------


OUTCOME VARIABLE:

 punish


              Coeff   BootMean     BootSE   BootLLCI   BootULCI

constant     3,9752     3,9784      ,3016     3,3858     4,5551

X1            ,1616      ,1569      ,1730     -,1799      ,5014

X2            ,1148      ,1140      ,1575     -,1982      ,4212

X3            ,0236      ,0216      ,1778     -,3305      ,3738

social_a     -,1178     -,1183      ,0335     -,1851     -,0520

Trans_s         ,9696      ,9703      ,1124      ,7532     1,1858


*********************** ANALYSIS NOTES AND ERRORS ************************


Level of confidence for all confidence intervals in output:

  95,0000


Number of bootstrap samples for percentile bootstrap confidence intervals:

  5000


NOTE: Variables names longer than eight characters can produce incorrect output.

      Shorter variable names are recommended.


------ END MATRIX -----

**Model6**

**M = Empathy // Y = Punishment**

Run MATRIX procedure:


**************** PROCESS Procedure for SPSS Version 3.1 ******************


          Written by Andrew F. Hayes, Ph.D.       www.afhayes.com

    Documentation available in Hayes (2018). www.guilford.com/p/hayes3


**************************************************************************

Model  : 4

    Y  : punish

    X  : Emotion

    M  : empathy


Covariates:

 Trans_s


Sample

Size:  924


Coding of categorical X variable for analysis:

 Emotion       X1       X2       X3

    1,000     ,000     ,000     ,000

    2,000    1,000     ,000     ,000

    3,000     ,000    1,000     ,000

    4,000     ,000     ,000    1,000


**************************************************************************

OUTCOME VARIABLE:

 empathy


Model Summary

          R       R-sq        MSE          F        df1        df2          p

      ,2982      ,0889     3,6172    22,4180     4,0000   919,0000      ,0000


Model

              coeff         se          t          p       LLCI       ULCI

constant     6,0848      ,2243    27,1266      ,0000     5,6446     6,5250

X1           -,7681      ,1738    -4,4204      ,0000    -1,1091     -,4271

X2           -,1076      ,1787     -,6019      ,5474     -,4583      ,2432

X3           -,9825      ,1752    -5,6066      ,0000    -1,3264     -,6386

Trans_s        -,8669      ,1267    -6,8417      ,0000    -1,1155     -,6182


Covariance matrix of regression parameter estimates:

           constant         X1         X2         X3      Trans_s

constant      ,0503     -,0161     -,0172     -,0167     -,0238

X1           -,0161      ,0302      ,0150      ,0149      ,0008

X2           -,0172      ,0150      ,0319      ,0150      ,0016

X3           -,0167      ,0149      ,0150      ,0307      ,0012

Trans_s        -,0238      ,0008      ,0016      ,0012      ,0161


**************************************************************************

OUTCOME VARIABLE:

 punish


Model Summary

          R       R-sq        MSE          F        df1        df2          p

      ,5066      ,2566     2,3530    63,3835     5,0000   918,0000      ,0000


Model

              coeff         se          t          p       LLCI       ULCI

constant     5,5555      ,2428    22,8837      ,0000     5,0791     6,0320

X1            ,0473      ,1416      ,3340      ,7384     -,2306      ,3252

X2            ,0872      ,1442      ,6049      ,5454     -,1957      ,3702

X3           -,0852      ,1437     -,5931      ,5533     -,3673      ,1968

empathy      -,3912      ,0266   -14,7027      ,0000     -,4434     -,3390

Trans_s         ,6544      ,1048     6,2465      ,0000      ,4488      ,8600


Covariance matrix of regression parameter estimates:

           constant         X1         X2         X3    empathy      Trans_s

constant      ,0589     -,0138     -,0117     -,0151     -,0043     -,0192

X1           -,0138      ,0201      ,0098      ,0103      ,0005      ,0010

X2           -,0117      ,0098      ,0208      ,0098      ,0001      ,0011

X3           -,0151      ,0103      ,0098      ,0207      ,0007      ,0014

empathy      -,0043      ,0005      ,0001      ,0007      ,0007      ,0006

Trans_s        -,0192      ,0010      ,0011      ,0014      ,0006      ,0110


************************** TOTAL EFFECT MODEL ****************************

OUTCOME VARIABLE:

 punish


Model Summary

          R       R-sq        MSE          F        df1        df2          p

      ,2856      ,0816     2,9040    20,4089     4,0000   919,0000      ,0000


Model

              coeff         se          t          p       LLCI       ULCI

constant     3,1753      ,2010    15,7991      ,0000     2,7809     3,5698

X1            ,3477      ,1557     2,2337      ,0257      ,0422      ,6533

X2            ,1293      ,1601      ,8073      ,4197     -,1850      ,4436

X3            ,2991      ,1570     1,9048      ,0571     -,0091      ,6072

Trans_s         ,9935      ,1135     8,7512      ,0000      ,7707     1,2163


Covariance matrix of regression parameter estimates:

           constant         X1         X2         X3      Trans_s

constant      ,0404     -,0129     -,0138     -,0134     -,0191

X1           -,0129      ,0242      ,0120      ,0120      ,0007

X2           -,0138      ,0120      ,0256      ,0120      ,0013

X3           -,0134      ,0120      ,0120      ,0247      ,0010

Trans_s        -,0191      ,0007      ,0013      ,0010      ,0129


************** TOTAL, DIRECT, AND INDIRECT EFFECTS OF X ON Y **************


Relative total effects of X on Y:

       Effect         se          t          p       LLCI       ULCI       c_ps

X1      ,3477      ,1557     2,2337      ,0257      ,0422      ,6533      ,1960

X2      ,1293      ,1601      ,8073      ,4197     -,1850      ,4436      ,0729

X3      ,2991      ,1570     1,9048      ,0571     -,0091      ,6072      ,1686


Omnibus test of total effect of X on Y:

    R2-chng          F        df1        df2          p

      ,0062     2,0843     3,0000   919,0000      ,1007

----------


Relative direct effects of X on Y

       Effect         se          t          p       LLCI       ULCI      c'_ps

X1      ,0473      ,1416      ,3340      ,7384     -,2306      ,3252      ,0267

X2      ,0872      ,1442      ,6049      ,5454     -,1957      ,3702      ,0491

X3     -,0852      ,1437     -,5931      ,5533     -,3673      ,1968     -,0480


Omnibus test of direct effect of X on Y:

    R2-chng          F        df1        df2          p

      ,0013      ,5188     3,0000   918,0000      ,6694

----------


Relative indirect effects of X on Y


 Emotion    ->    empathy     ->    punish


       Effect     BootSE   BootLLCI   BootULCI

X1      ,3004      ,0712      ,1615      ,4426

X2      ,0421      ,0702     -,0975      ,1792

X3      ,3843      ,0748      ,2379      ,5320


Partially standardized relative indirect effect(s) of X on Y:


 Emotion    ->    empathy     ->    punish


       Effect     BootSE   BootLLCI   BootULCI

X1      ,1693      ,0399      ,0915      ,2489

X2      ,0237      ,0396     -,0551      ,1012

X3      ,2166      ,0417      ,1350      ,2994


*********** BOOTSTRAP RESULTS FOR REGRESSION MODEL PARAMETERS ************


OUTCOME VARIABLE:

 empathy


              Coeff   BootMean     BootSE   BootLLCI   BootULCI

constant     6,0848     6,0840      ,2177     5,6564     6,5122

X1           -,7681     -,7672      ,1739    -1,1042     -,4176

X2           -,1076     -,1055      ,1787     -,4526      ,2479

X3           -,9825     -,9783      ,1777    -1,3223     -,6158

Trans_s        -,8669     -,8673      ,1254    -1,1132     -,6198


----------


OUTCOME VARIABLE:

 punish


              Coeff   BootMean     BootSE   BootLLCI   BootULCI

constant     5,5555     5,5557      ,2563     5,0481     6,0490

X1            ,0473      ,0470      ,1479     -,2414      ,3324

X2            ,0872      ,0870      ,1437     -,1942      ,3686

X3           -,0852     -,0856      ,1454     -,3707      ,1954

empathy      -,3912     -,3912      ,0275     -,4437     -,3366

Trans_s         ,6544      ,6558      ,1077      ,4517      ,8682


*********************** ANALYSIS NOTES AND ERRORS ************************


Level of confidence for all confidence intervals in output:

  95,0000


Number of bootstrap samples for percentile bootstrap confidence intervals:

  5000


------ END MATRIX -----

**PARALLEL MEDIATION MODELS**

**Model7**

**Y = Cooperation**

Run MATRIX procedure:


**************** PROCESS Procedure for SPSS Version 3.1 ******************


          Written by Andrew F. Hayes, Ph.D.       www.afhayes.com

    Documentation available in Hayes (2018). www.guilford.com/p/hayes3


**************************************************************************

Model  : 4

    Y  : cooper

    X  : Emotion

   M1  : empathy

   M2  : moral_s

   M3  : social_a


Covariates:

 Trans_s


Sample

Size:  924


Coding of categorical X variable for analysis:

 Emotion       X1       X2       X3

    1,000     ,000     ,000     ,000

    2,000    1,000     ,000     ,000

    3,000     ,000    1,000     ,000

    4,000     ,000     ,000    1,000


**************************************************************************

OUTCOME VARIABLE:

 empathy


Model Summary

          R       R-sq        MSE          F        df1        df2          p

      ,2982      ,0889     3,6172    22,4180     4,0000   919,0000      ,0000


Model

              coeff         se          t          p       LLCI       ULCI

constant     6,0848      ,2243    27,1266      ,0000     5,6446     6,5250

X1           -,7681      ,1738    -4,4204      ,0000    -1,1091     -,4271

X2           -,1076      ,1787     -,6019      ,5474     -,4583      ,2432

X3           -,9825      ,1752    -5,6066      ,0000    -1,3264     -,6386

Trans_s        -,8669      ,1267    -6,8417      ,0000    -1,1155     -,6182


Covariance matrix of regression parameter estimates:

           constant         X1         X2         X3      Trans_s

constant      ,0503     -,0161     -,0172     -,0167     -,0238

X1           -,0161      ,0302      ,0150      ,0149      ,0008

X2           -,0172      ,0150      ,0319      ,0150      ,0016

X3           -,0167      ,0149      ,0150      ,0307      ,0012

Trans_s        -,0238      ,0008      ,0016      ,0012      ,0161


**************************************************************************

OUTCOME VARIABLE:

 moral_s


Model Summary

          R       R-sq        MSE          F        df1        df2          p

      ,4878      ,2379     2,5887    71,7365     4,0000   919,0000      ,0000


Model

              coeff         se          t          p       LLCI       ULCI

constant     6,0989      ,1898    32,1408      ,0000     5,7265     6,4714

X1          -1,8765      ,1470   -12,7661      ,0000    -2,1649    -1,5880

X2           -,3657      ,1512    -2,4185      ,0158     -,6624     -,0689

X3          -2,0051      ,1482   -13,5261      ,0000    -2,2961    -1,7142

Trans_s        -,1816      ,1072    -1,6939      ,0906     -,3919      ,0288


Covariance matrix of regression parameter estimates:

           constant         X1         X2         X3      Trans_s

constant      ,0360     -,0115     -,0123     -,0120     -,0171

X1           -,0115      ,0216      ,0107      ,0107      ,0006

X2           -,0123      ,0107      ,0229      ,0107      ,0011

X3           -,0120      ,0107      ,0107      ,0220      ,0009

Trans_s        -,0171      ,0006      ,0011      ,0009      ,0115


**************************************************************************

OUTCOME VARIABLE:

 social_a


Model Summary

          R       R-sq        MSE          F        df1        df2          p

      ,4987      ,2487     2,9707    76,0588     4,0000   919,0000      ,0000


Model

              coeff         se          t          p       LLCI       ULCI

constant     6,7903      ,2033    33,4038      ,0000     6,3914     7,1892

X1          -1,5802      ,1575   -10,0354      ,0000    -1,8892    -1,2712

X2           -,1232      ,1620     -,7606      ,4471     -,4411      ,1947

X3          -2,3386      ,1588   -14,7262      ,0000    -2,6503    -2,0269

Trans_s        -,2023      ,1148    -1,7621      ,0784     -,4277      ,0230


Covariance matrix of regression parameter estimates:

           constant         X1         X2         X3      Trans_s

constant      ,0413     -,0132     -,0141     -,0137     -,0196

X1           -,0132      ,0248      ,0123      ,0123      ,0007

X2           -,0141      ,0123      ,0262      ,0123      ,0013

X3           -,0137      ,0123      ,0123      ,0252      ,0010

Trans_s        -,0196      ,0007      ,0013      ,0010      ,0132


**************************************************************************

OUTCOME VARIABLE:

 cooper


Model Summary

          R       R-sq        MSE          F        df1        df2          p

      ,6033      ,3640     2,4815    74,8775     7,0000   916,0000      ,0000


Model

              coeff         se          t          p       LLCI       ULCI

constant     1,8730      ,3027     6,1881      ,0000     1,2790     2,4670

X1           -,0967      ,1569     -,6163      ,5378     -,4046      ,2112

X2           -,1576      ,1486    -1,0606      ,2891     -,4492      ,1340

X3           -,0558      ,1637     -,3405      ,7335     -,3771      ,2656

empathy       ,4621      ,0304    15,1979      ,0000      ,4024      ,5217

moral_s      ,2074      ,0431     4,8163      ,0000      ,1229      ,2919

social_a      ,0146      ,0381      ,3824      ,7023     -,0602      ,0893

Trans_s        -,1897      ,1077    -1,7611      ,0786     -,4011      ,0217


Covariance matrix of regression parameter estimates:

           constant         X1         X2         X3    empathy   moral_s   social_a      Trans_s

constant      ,0916     -,0239     -,0135     -,0281     -,0025     -,0025     -,0039     -,0198

X1           -,0239      ,0246      ,0109      ,0147     -,0002      ,0017      ,0005      ,0008

X2           -,0135      ,0109      ,0221      ,0109     -,0001      ,0005     -,0002      ,0011

X3           -,0281      ,0147      ,0109      ,0268     -,0001      ,0012      ,0015      ,0013

empathy      -,0025     -,0002     -,0001     -,0001      ,0009     -,0004     -,0001      ,0007

moral_s     -,0025      ,0017      ,0005      ,0012     -,0004      ,0019     -,0009     -,0002

social_a     -,0039      ,0005     -,0002      ,0015     -,0001     -,0009      ,0014      ,0001

Trans_s        -,0198      ,0008      ,0011      ,0013      ,0007     -,0002      ,0001      ,0116


************************** TOTAL EFFECT MODEL ****************************

OUTCOME VARIABLE:

 cooper


Model Summary

          R       R-sq        MSE          F        df1        df2          p

      ,2546      ,0648     3,6367    15,9264     4,0000   919,0000      ,0000


Model

              coeff         se          t          p       LLCI       ULCI

constant     6,0483      ,2249    26,8917      ,0000     5,6069     6,4897

X1           -,8637      ,1742    -4,9578      ,0000    -1,2057     -,5218

X2           -,2849      ,1792    -1,5899      ,1122     -,6366      ,0668

X3           -,9596      ,1757    -5,4615      ,0000    -1,3045     -,6148

Trans_s        -,6308      ,1270    -4,9656      ,0000     -,8802     -,3815


Covariance matrix of regression parameter estimates:

           constant         X1         X2         X3      Trans_s

constant      ,0506     -,0162     -,0173     -,0168     -,0240

X1           -,0162      ,0304      ,0150      ,0150      ,0008

X2           -,0173      ,0150      ,0321      ,0151      ,0016

X3           -,0168      ,0150      ,0151      ,0309      ,0012

Trans_s        -,0240      ,0008      ,0016      ,0012      ,0161


************** TOTAL, DIRECT, AND INDIRECT EFFECTS OF X ON Y **************


Relative total effects of X on Y:

       Effect         se          t          p       LLCI       ULCI       c_ps

X1     -,8637      ,1742    -4,9578      ,0000    -1,2057     -,5218     -,4390

X2     -,2849      ,1792    -1,5899      ,1122     -,6366      ,0668     -,1448

X3     -,9596      ,1757    -5,4615      ,0000    -1,3045     -,6148     -,4877


Omnibus test of total effect of X on Y:

    R2-chng          F        df1        df2          p

      ,0419    13,7263     3,0000   919,0000      ,0000

----------


Relative direct effects of X on Y

       Effect         se          t          p       LLCI       ULCI      c'_ps

X1     -,0967      ,1569     -,6163      ,5378     -,4046      ,2112     -,0491

X2     -,1576      ,1486    -1,0606      ,2891     -,4492      ,1340     -,0801

X3     -,0558      ,1637     -,3405      ,7335     -,3771      ,2656     -,0283


Omnibus test of direct effect of X on Y:

    R2-chng          F        df1        df2          p

      ,0008      ,4001     3,0000   916,0000      ,7530

----------


Relative indirect effects of X on Y


 Emotion    ->    empathy     ->    cooper


       Effect     BootSE   BootLLCI   BootULCI

X1     -,3549      ,0849     -,5257     -,1945

X2     -,0497      ,0860     -,2180      ,1191

X3     -,4540      ,0899     -,6298     -,2826


 Emotion    ->    moral_s    ->    cooper


       Effect     BootSE   BootLLCI   BootULCI

X1     -,3891      ,0933     -,5812     -,2176

X2     -,0758      ,0363     -,1563     -,0146

X3     -,4158      ,0989     -,6226     -,2336


 Emotion    ->    social_a    ->    cooper


       Effect     BootSE   BootLLCI   BootULCI

X1     -,0230      ,0668     -,1524      ,1075

X2     -,0018      ,0085     -,0206      ,0152

X3     -,0340      ,0989     -,2283      ,1611


Partially standardized relative indirect effect(s) of X on Y:


 Emotion    ->    empathy     ->    cooper


       Effect     BootSE   BootLLCI   BootULCI

X1     -,1804      ,0425     -,2648     -,0986

X2     -,0253      ,0437     -,1099      ,0604

X3     -,2307      ,0447     -,3192     -,1447


 Emotion    ->    moral_s    ->    cooper


       Effect     BootSE   BootLLCI   BootULCI

X1     -,1978      ,0472     -,2959     -,1111

X2     -,0385      ,0184     -,0792     -,0075

X3     -,2113      ,0500     -,3156     -,1191


 Emotion    ->    social_a    ->    cooper


       Effect     BootSE   BootLLCI   BootULCI

X1     -,0117      ,0340     -,0776      ,0550

X2     -,0009      ,0043     -,0105      ,0078

X3     -,0173      ,0503     -,1160      ,0820


*********** BOOTSTRAP RESULTS FOR REGRESSION MODEL PARAMETERS ************


OUTCOME VARIABLE:

 empathy


              Coeff   BootMean     BootSE   BootLLCI   BootULCI

constant     6,0848     6,0889      ,2222     5,6561     6,5193

X1           -,7681     -,7706      ,1754    -1,1125     -,4256

X2           -,1076     -,1101      ,1856     -,4702      ,2543

X3           -,9825     -,9834      ,1791    -1,3313     -,6349

Trans_s        -,8669     -,8676      ,1267    -1,1102     -,6159


----------


OUTCOME VARIABLE:

 moral_s


              Coeff   BootMean     BootSE   BootLLCI   BootULCI

constant     6,0989     6,0987      ,1875     5,7208     6,4609

X1          -1,8765    -1,8762      ,1445    -2,1604    -1,5967

X2           -,3657     -,3677      ,1510     -,6662     -,0773

X3          -2,0051    -2,0044      ,1468    -2,2877    -1,7212

Trans_s        -,1816     -,1808      ,1082     -,3911      ,0341


----------


OUTCOME VARIABLE:

 social_a


              Coeff   BootMean     BootSE   BootLLCI   BootULCI

constant     6,7903     6,7871      ,1993     6,3902     7,1723

X1          -1,5802    -1,5764      ,1600    -1,8917    -1,2621

X2           -,1232     -,1226      ,1549     -,4274      ,1819

X3          -2,3386    -2,3370      ,1557    -2,6447    -2,0390

Trans_s        -,2023     -,2008      ,1157     -,4254      ,0305


----------


OUTCOME VARIABLE:

 cooper


              Coeff   BootMean     BootSE   BootLLCI   BootULCI

constant     1,8730     1,8695      ,3113     1,2579     2,4766

X1           -,0967     -,0982      ,1583     -,4115      ,2225

X2           -,1576     -,1578      ,1500     -,4572      ,1297

X3           -,0558     -,0559      ,1651     -,3803      ,2756

empathy       ,4621      ,4617      ,0332      ,3954      ,5263

moral_s      ,2074      ,2095      ,0464      ,1189      ,3006

social_a      ,0146      ,0134      ,0422     -,0672      ,0972

Trans_s        -,1897     -,1883      ,1087     -,4006      ,0234


*********************** ANALYSIS NOTES AND ERRORS ************************


Level of confidence for all confidence intervals in output:

  95,0000


Number of bootstrap samples for percentile bootstrap confidence intervals:

  5000


NOTE: Variables names longer than eight characters can produce incorrect output.

      Shorter variable names are recommended.


------ END MATRIX -----

**Model8**

**Y = Punishment**

Run MATRIX procedure:


**************** PROCESS Procedure for SPSS Version 3.1 ******************


          Written by Andrew F. Hayes, Ph.D.       www.afhayes.com

    Documentation available in Hayes (2018). www.guilford.com/p/hayes3


**************************************************************************

Model  : 4

    Y  : punish

    X  : Emotion

   M1  : empathy

   M2  : moral_s

   M3  : social_a


Covariates:

 Trans_s


Sample

Size:  924


Coding of categorical X variable for analysis:

 Emotion       X1       X2       X3

    1,000     ,000     ,000     ,000

    2,000    1,000     ,000     ,000

    3,000     ,000    1,000     ,000

    4,000     ,000     ,000    1,000


**************************************************************************

OUTCOME VARIABLE:

 empathy


Model Summary

          R       R-sq        MSE          F        df1        df2          p

      ,2982      ,0889     3,6172    22,4180     4,0000   919,0000      ,0000


Model

              coeff         se          t          p       LLCI       ULCI

constant     6,0848      ,2243    27,1266      ,0000     5,6446     6,5250

X1           -,7681      ,1738    -4,4204      ,0000    -1,1091     -,4271

X2           -,1076      ,1787     -,6019      ,5474     -,4583      ,2432

X3           -,9825      ,1752    -5,6066      ,0000    -1,3264     -,6386

Trans_s        -,8669      ,1267    -6,8417      ,0000    -1,1155     -,6182


Covariance matrix of regression parameter estimates:

           constant         X1         X2         X3      Trans_s

constant      ,0503     -,0161     -,0172     -,0167     -,0238

X1           -,0161      ,0302      ,0150      ,0149      ,0008

X2           -,0172      ,0150      ,0319      ,0150      ,0016

X3           -,0167      ,0149      ,0150      ,0307      ,0012

Trans_s        -,0238      ,0008      ,0016      ,0012      ,0161


**************************************************************************

OUTCOME VARIABLE:

 moral_s


Model Summary

          R       R-sq        MSE          F        df1        df2          p

      ,4878      ,2379     2,5887    71,7365     4,0000   919,0000      ,0000


Model

              coeff         se          t          p       LLCI       ULCI

constant     6,0989      ,1898    32,1408      ,0000     5,7265     6,4714

X1          -1,8765      ,1470   -12,7661      ,0000    -2,1649    -1,5880

X2           -,3657      ,1512    -2,4185      ,0158     -,6624     -,0689

X3          -2,0051      ,1482   -13,5261      ,0000    -2,2961    -1,7142

Trans_s        -,1816      ,1072    -1,6939      ,0906     -,3919      ,0288


Covariance matrix of regression parameter estimates:

           constant         X1         X2         X3      Trans_s

constant      ,0360     -,0115     -,0123     -,0120     -,0171

X1           -,0115      ,0216      ,0107      ,0107      ,0006

X2           -,0123      ,0107      ,0229      ,0107      ,0011

X3           -,0120      ,0107      ,0107      ,0220      ,0009

Trans_s        -,0171      ,0006      ,0011      ,0009      ,0115


**************************************************************************

OUTCOME VARIABLE:

 social_a


Model Summary

          R       R-sq        MSE          F        df1        df2          p

      ,4987      ,2487     2,9707    76,0588     4,0000   919,0000      ,0000


Model

              coeff         se          t          p       LLCI       ULCI

constant     6,7903      ,2033    33,4038      ,0000     6,3914     7,1892

X1          -1,5802      ,1575   -10,0354      ,0000    -1,8892    -1,2712

X2           -,1232      ,1620     -,7606      ,4471     -,4411      ,1947

X3          -2,3386      ,1588   -14,7262      ,0000    -2,6503    -2,0269

Trans_s        -,2023      ,1148    -1,7621      ,0784     -,4277      ,0230


Covariance matrix of regression parameter estimates:

           constant         X1         X2         X3      Trans_s

constant      ,0413     -,0132     -,0141     -,0137     -,0196

X1           -,0132      ,0248      ,0123      ,0123      ,0007

X2           -,0141      ,0123      ,0262      ,0123      ,0013

X3           -,0137      ,0123      ,0123      ,0252      ,0010

Trans_s        -,0196      ,0007      ,0013      ,0010      ,0132


**************************************************************************

OUTCOME VARIABLE:

 punish


Model Summary

          R       R-sq        MSE          F        df1        df2          p

      ,5160      ,2663     2,3276    47,4916     7,0000   916,0000      ,0000


Model

              coeff         se          t          p       LLCI       ULCI

constant     5,6611      ,2931    19,3123      ,0000     5,0858     6,2364

X1           -,0606      ,1519     -,3985      ,6903     -,3588      ,2376

X2            ,0488      ,1439      ,3392      ,7345     -,2336      ,3312

X3           -,1390      ,1586     -,8765      ,3810     -,4502      ,1722

empathy      -,3633      ,0294   -12,3387      ,0000     -,4211     -,3055

moral_s     -,1427      ,0417    -3,4226      ,0006     -,2246     -,0609

social_a      ,0877      ,0369     2,3782      ,0176      ,0153      ,1601

Trans_s         ,6704      ,1043     6,4264      ,0000      ,4656      ,8751


Covariance matrix of regression parameter estimates:

           constant         X1         X2         X3    empathy   moral_s   social_a      Trans_s

constant      ,0859     -,0224     -,0127     -,0263     -,0024     -,0024     -,0036     -,0186

X1           -,0224      ,0231      ,0103      ,0138     -,0002      ,0016      ,0005      ,0008

X2           -,0127      ,0103      ,0207      ,0102     -,0001      ,0005     -,0002      ,0010

X3           -,0263      ,0138      ,0102      ,0251     -,0001      ,0011      ,0014      ,0012

empathy      -,0024     -,0002     -,0001     -,0001      ,0009     -,0004     -,0001      ,0007

moral_s     -,0024      ,0016      ,0005      ,0011     -,0004      ,0017     -,0009     -,0002

social_a     -,0036      ,0005     -,0002      ,0014     -,0001     -,0009      ,0014      ,0001

Trans_s        -,0186      ,0008      ,0010      ,0012      ,0007     -,0002      ,0001      ,0109


************************** TOTAL EFFECT MODEL ****************************

OUTCOME VARIABLE:

 punish


Model Summary

          R       R-sq        MSE          F        df1        df2          p

      ,2856      ,0816     2,9040    20,4089     4,0000   919,0000      ,0000


Model

              coeff         se          t          p       LLCI       ULCI

constant     3,1753      ,2010    15,7991      ,0000     2,7809     3,5698

X1            ,3477      ,1557     2,2337      ,0257      ,0422      ,6533

X2            ,1293      ,1601      ,8073      ,4197     -,1850      ,4436

X3            ,2991      ,1570     1,9048      ,0571     -,0091      ,6072

Trans_s         ,9935      ,1135     8,7512      ,0000      ,7707     1,2163


Covariance matrix of regression parameter estimates:

           constant         X1         X2         X3      Trans_s

constant      ,0404     -,0129     -,0138     -,0134     -,0191

X1           -,0129      ,0242      ,0120      ,0120      ,0007

X2           -,0138      ,0120      ,0256      ,0120      ,0013

X3           -,0134      ,0120      ,0120      ,0247      ,0010

Trans_s        -,0191      ,0007      ,0013      ,0010      ,0129


************** TOTAL, DIRECT, AND INDIRECT EFFECTS OF X ON Y **************


Relative total effects of X on Y:

       Effect         se          t          p       LLCI       ULCI       c_ps

X1      ,3477      ,1557     2,2337      ,0257      ,0422      ,6533      ,1960

X2      ,1293      ,1601      ,8073      ,4197     -,1850      ,4436      ,0729

X3      ,2991      ,1570     1,9048      ,0571     -,0091      ,6072      ,1686


Omnibus test of total effect of X on Y:

    R2-chng          F        df1        df2          p

      ,0062     2,0843     3,0000   919,0000      ,1007

----------


Relative direct effects of X on Y

       Effect         se          t          p       LLCI       ULCI      c'_ps

X1     -,0606      ,1519     -,3985      ,6903     -,3588      ,2376     -,0341

X2      ,0488      ,1439      ,3392      ,7345     -,2336      ,3312      ,0275

X3     -,1390      ,1586     -,8765      ,3810     -,4502      ,1722     -,0783


Omnibus test of direct effect of X on Y:

    R2-chng          F        df1        df2          p

      ,0012      ,4838     3,0000   916,0000      ,6936

----------


Relative indirect effects of X on Y


 Emotion    ->    empathy     ->    punish


       Effect     BootSE   BootLLCI   BootULCI

X1      ,2790      ,0687      ,1496      ,4161

X2      ,0391      ,0668     -,0892      ,1695

X3      ,3570      ,0733      ,2184      ,5073


 Emotion    ->    moral_s    ->    punish


       Effect     BootSE   BootLLCI   BootULCI

X1      ,2678      ,0853      ,1023      ,4413

X2      ,0522      ,0279      ,0076      ,1148

X3      ,2862      ,0894      ,1111      ,4608


 Emotion    ->    social_a    ->    punish


       Effect     BootSE   BootLLCI   BootULCI

X1     -,1386      ,0598     -,2575     -,0225

X2     -,0108      ,0153     -,0447      ,0180

X3     -,2051      ,0885     -,3818     -,0329


Partially standardized relative indirect effect(s) of X on Y:


 Emotion    ->    empathy     ->    punish


       Effect     BootSE   BootLLCI   BootULCI

X1      ,1573      ,0385      ,0842      ,2332

X2      ,0220      ,0377     -,0501      ,0960

X3      ,2012      ,0408      ,1234      ,2856


 Emotion    ->    moral_s    ->    punish


       Effect     BootSE   BootLLCI   BootULCI

X1      ,1509      ,0479      ,0582      ,2470

X2      ,0294      ,0157      ,0043      ,0643

X3      ,1613      ,0503      ,0624      ,2589


 Emotion    ->    social_a    ->    punish


       Effect     BootSE   BootLLCI   BootULCI

X1     -,0781      ,0336     -,1444     -,0128

X2     -,0061      ,0086     -,0251      ,0101

X3     -,1156      ,0497     -,2144     -,0188


*********** BOOTSTRAP RESULTS FOR REGRESSION MODEL PARAMETERS ************


OUTCOME VARIABLE:

 empathy


              Coeff   BootMean     BootSE   BootLLCI   BootULCI

constant     6,0848     6,0868      ,2186     5,6609     6,5114

X1           -,7681     -,7641      ,1740    -1,1064     -,4185

X2           -,1076     -,1062      ,1830     -,4652      ,2463

X3           -,9825     -,9836      ,1797    -1,3336     -,6335

Trans_s        -,8669     -,8680      ,1233    -1,1150     -,6245


----------


OUTCOME VARIABLE:

 moral_s


              Coeff   BootMean     BootSE   BootLLCI   BootULCI

constant     6,0989     6,1011      ,1886     5,7343     6,4713

X1          -1,8765    -1,8775      ,1414    -2,1572    -1,5964

X2           -,3657     -,3644      ,1486     -,6571     -,0741

X3          -2,0051    -2,0067      ,1474    -2,3032    -1,7236

Trans_s        -,1816     -,1825      ,1096     -,3981      ,0291


----------


OUTCOME VARIABLE:

 social_a


              Coeff   BootMean     BootSE   BootLLCI   BootULCI

constant     6,7903     6,7906      ,2017     6,4018     7,1856

X1          -1,5802    -1,5793      ,1598    -1,8928    -1,2634

X2           -,1232     -,1238      ,1545     -,4307      ,1859

X3          -2,3386    -2,3370      ,1568    -2,6456    -2,0223

Trans_s        -,2023     -,2029      ,1170     -,4368      ,0285


----------


OUTCOME VARIABLE:

 punish


              Coeff   BootMean     BootSE   BootLLCI   BootULCI

constant     5,6611     5,6614      ,3079     5,0537     6,2611

X1           -,0606     -,0589      ,1577     -,3627      ,2483

X2            ,0488      ,0498      ,1450     -,2319      ,3287

X3           -,1390     -,1371      ,1648     -,4559      ,1902

empathy      -,3633     -,3640      ,0319     -,4244     -,2998

moral_s     -,1427     -,1412      ,0434     -,2255     -,0546

social_a      ,0877      ,0869      ,0374      ,0144      ,1612

Trans_s         ,6704      ,6699      ,1061      ,4613      ,8779


*********************** ANALYSIS NOTES AND ERRORS ************************


Level of confidence for all confidence intervals in output:

  95,0000


Number of bootstrap samples for percentile bootstrap confidence intervals:

  5000


NOTE: Variables names longer than eight characters can produce incorrect output.

      Shorter variable names are recommended.

------ END MATRIX -----
